# ACO1 and IREB2 downregulation confer poor prognosis and correlate with autophagy-related ferroptosis and immune infiltration in KIRC

**DOI:** 10.3389/fonc.2022.929838

**Published:** 2022-08-17

**Authors:** Ting Zhu, Zhuoyu Xiao, Haoyu Yuan, Hu Tian, Taoyi Chen, Qi Chen, Mingkun Chen, Jiankun Yang, Qizhao Zhou, Wenbin Guo, Kangyi Xue, Ming Xia, Jiming Bao, Cheng Yang, Haifeng Duan, Hongyi Wang, Zhipeng Huang, Cundong Liu, Junhao Zhou

**Affiliations:** ^1^ Department of Laboratory Medicine, Affiliated Cancer Hospital and Institute of Guangzhou Medical University, Guangzhou, China; ^2^ Department of Urology, The Third Affiliated Hospital of Southern Medical University, Guangzhou, China

**Keywords:** ACO1, IREB2, kidney renal clear cell carcinoma, ferroptosis, disease progression, tumor immune infiltration

## Abstract

**Background:**

ACO1 and IREB2 are two homologous cytosolic regulatory proteins, which sense iron levels and change iron metabolism–linked molecules. These two genes were noticeably decreased in kidney renal clear cell carcinoma (KIRC), which confer poor survival. Meanwhile, there is a paucity of information about the mechanisms and clinical significance of ACO1 and IREB2 downregulation in renal cancers.

**Methods:**

The expression profiles of ACO1 and IREB2 were assessed using multiple public data sets *via* several bioinformatics platforms. Clinical and pathological information was utilized to stratify cohorts for comparison. Patient survival outcomes were evaluated using the Kaplan–Meier plotter, a meta-analysis tool. The correlations of ACO1 and IREB2 with ferroptosis were further evaluated in The Cancer Genome Atlas (TCGA)–KIRC database. Tumor immune infiltration was analyzed using the CIBERSORT, TIMER, and GEPIA data resources. ACO1 antagonist sodium oxalomalate (OMA) and IREB2 inhibitor sodium nitroprusside (SNP) was used to treat renal cancer ACHN cells together with sorafenib.

**Results:**

KIRC patients with low ACO1 or IREB2 contents exhibited a remarkably worse survival rate in contrast with those with high expression in Kaplan–Meier survival analyses. Meanwhile, ACO1 and IREB2 regulate autophagy-linked ferroptosis along with immune cell invasion in the tumor microenvironment in KIRC patients. Blocking the activation of these two genes by their inhibitors OMA and SNP ameliorated sorafenib-triggered cell death, supporting that ACO1 and IREB2 could be participated in its cytotoxic influence on renal cancer cells.

**Conclusion:**

ACO1 and IREB2 downregulation in renal cancers were correlated with cancer aggressiveness, cellular iron homeostasis, cytotoxic immune cell infiltration, and patient survival outcomes. Our research is integral to verify the possible significance of ACO1 and IREB2 contents as a powerful signature for targeted treatment or novel immunotherapy in clinical settings.

## Introduction

Renal cell carcinoma (RCC) accounts for approximately 3% of all human systemic malignancies and 85%–90% of all primary malignancies in the adult kidney (1). New cases of RCC were reported to be 66,800 in 2015 (43,200 for men and 23,600 for women), accounting for 1.56% malignancy cases and ranked 14th among all malignancy cases. RCC was linked to 23,400 deaths (15,200 men and 8,200 women), accounting for 0.83% of all cancer fatalities and ranked 17th among all cancer deaths (2). Despite remarkable advancements in surgical treatments and targeted therapeutic medications, all of the existing treatments for individuals with renal malignant tumor strive to attain acceptable survival rates (3). For renal malignant tumor, there is a pressing need to explore effective prognostic markers and novel powerful treatment targets. The most prevalent histological kind of RCC, KIRC, was investigated further in this research.

Iron proved to be one of the most plentiful trace elements, which works an essential role for cell functions (4). The deficiency of iron may lead to growth arrest and even cell death; meanwhile, iron overload produces free radicals that can cause harm to DNA, membranes, and proteins (5). The dysregulations of iron homeostasis have gained great attention as a vital mechanism in tumorigenesis (5–7). The maintenance of the cellular and systematic homeostasis of iron is mainly modulated by iron modulatory proteins (IRPs: IRP1 and IRP2), which are sensitized in iron-underprovided conditions (8). IRP1 (also known as IREB1 and ACO1) and IRP2 (also known as IREB2) are two homologous cytosolic regulatory proteins. By binding to iron-responsive elements (IREs) inside the target transcripts, these two proteins sense iron levels in the cytosol and post-transcriptionally change iron metabolism-linked genes, for instance, transferrin receptor 1 (TfR1) along with the ferritin H and L subunits (9, 10). IRP1 is a powerful bifunctional enzyme that also works as a cytosolic aconitase (ACO1) (11). Once intracellular iron expression is higher than normal, ACO1 links to the iron–sulfur (4Fe-4S) cluster and works as an aconitase; once intracellular iron level turns lower, IRP1 works as an iron regulatory protein after it departs from the iron-sulfur cluster (12, 13). IRP1 is not sensitive to cellular iron levels but susceptible to the slight change of oxygen, nitrogen oxides, and hydroxides. ACO1 can be converted to IRP1 when oxygen concentrations increase, and its RNA-binding activity can significantly increase (14–16). On the contrary, IRP2 is susceptible to iron levels and turn to be activated when the cellular iron is underprovided (9). When IRP1 and IRP2 become active, they all dock to the 5’- UTR of the iron exporter protein ferroportin (FPN), as well as the iron storage protein ferritin, to prevent them from undergoing translation, reducing iron export along with storage (14, 17). Furthermore, IRP1 along with IRP2 can dock to the 3’-UTR of the transferrin receptor (TFRC), which participates in iron absorption, and prevents it from being degraded, hence augmenting iron uptake and maintaining intracellular iron homeostasis (14, 17).

An increasing number of studies reported that the maladjustment of iron homeostasis is the representative metabolic hallmark of cancer cells, and iron is essential for tumor occurrence, development, and metastasis (7, 18). Consistently, multiple studies have shown the ectopic expression of ACO1 and IREB2 in several types of solid cancer, including lung cancer, breast cancer, and prostate cancer. In human lung cancer cells, IRP1 overexpression significantly reduced the capacity of cancer cells to form tumor xenografts in nude mice (19). Both IRP1 and IRP2 are overexpressed in breast cancer. The knockdown of IRP2 significantly inhibits solid tumor growth whether internal or external, which significantly prolongs the survivals of cancer cells and tumor-bearing mice (20). In hepatoma cells, nitric oxide boosts IRE binding to IRP1 powerfully, but it plays a slight role when IRE binds to IRP2, suggesting the function for IRP1 in the modulation of iron homeostasis *in vivo* when suffering hepatic inflammation (21). Slowing the ACO1 vitality decreased the capability of T-cell lymphoblastic neoplasia cells, and ACO1 knockdown makes resistant cell lines susceptible to fluorocitrate, which represented that ACO1 is a new powerful therapeutic strategy for the healing of diverse cancers (22). The inhibition of IRP1 activity by SIRT3 represses the proliferation of human pancreatic cancer cells (23). The disorder of IRP1-mediated iron metabolism turns leukemia cells specifically resistant to gamma rays (24). In melanoma, IRP1 balances cellular iron homeostasis to drive ferroptosis (25). IRP2 promotes cell growth and drives its oncogenic activities by repressing TAp63 (26). IRP2 works as a suppressor of mutant p53 in tumorigenesis (27). IRP2 is overexpressed in prostate cancer cells, and its knockdown decreases intracellular iron levels and drives cell cycle arrest even in apoptosis (28). In addition, ACO1 and IREB2 were identified to be required for erastin-induced ferroptosis in lung cancer cells (29, 30). Therefore, ACO1 and IREB2 can work as independent risk factors and prognostic biomarkers of several types of cancer. Meanwhile, the function of ACO1 and IREB2 in KIRC progression and its relevance with ferroptosis in KIRC are less well understood.

Ferroptosis constitutes a non-apoptotic cell death characterized by a dependency on cellular iron ions and the aggregation of lipid ROS (30). Sorafenib is used for progressive kidney and liver cancer as a multikinase inhibitor. Sorafenib can induce ferroptosis *via* preventing the activity of the glutamate/cystine antiporter system Xc−, which carries cystine into cells (4, 31, 32). Reduced cystine consumption represses glutathione generation, lipid ROS buildup, and the onset of ferroptosis (30). The typical markers of ferroptosis constitute relative intracellular iron levels along with lipid ROS (33). Ferroptosis involves a number of genes that impact iron metabolism. Transferrin, for instance, is required for ferroptosis, and knocking out the TFRC gene can remarkably diminish ferroptosis (34). Moreover, ferritinophagy is a powerful launcher of ferroptosis. Ferritin heavy chain 1 (FTH1) is downregulated by autophagy throughout this process, resulting in intracellular iron aggregation and ferroptosis (35–37).

Weak evidence has revealed the role and clinical aspects of ACO1 and IREB2 in renal cancer at various stages of etiology and prognosis based on the strong relationship between iron homeostasis and carcinogenesis. The current work focuses on combining several bioinformatics tools to assess if ACO1 and IREB2 are involved in renal cancer development and ferroptosis, as well as their molecular regulation. When comparing KIRC tissues to healthy tissues, we established that ACO1 along with IREB2 contents were remarkably lower. Additionally, as tumor stages and distant metastasis progressed, ACO1 coupled with IREB2 contents decreased. The weak expression of IREBs was linked to a poor prognosis in individuals with KIRC. There were also substantial correlations between ACO1, IREB2, and autophagy-linked ferroptosis expression. Furthermore, in KIRC, there was a remarkable link between ACO1, IREB2, and the numbers of infiltrating B cells, CD4+ T cells, macrophages, CD8+ T cells, and neutrophils along with dendritic cells. Importantly, ACO1 along with IREB2 appeared to alter the prognosis of individuals with KIRC in part *via* infiltrating immune cells. These findings highlight ACO1 and IREB2’s important involvement in carcinogenesis, as well as their potential roles in modulating autophagy-linked ferroptosis and immune cell invasion in KIRC.

## Materials and methods

### Cell lines, culture condition, and experimental reagents

We purchased renal cancer cell lines ACHN from the American Type Culture Collection (Manassas, VA, USA). ACHN cells were cultured in Eagle’s Minimum Essential Medium enriched with 10% Fetal Bovine Serum (FBS) along with 1% penicillin/streptomycin under 37°C along with 5% CO_2_ conditions.

G-CLONE supplied the small chemicals sorafenib (GS0220) along with sodium nitroprusside (GN0200).

Sigma-Aldrich provided us with rapamycin (V900930). ABCAM supplied antibodies to ACSL4 (ab227256), beta-tubulin (ab6046), Beclin-1 (ab210498), LC3 (ab192890), FTH (ab183781), ATG12 (ab155589), and NCOA4 (ab86707). Secondary antibodies linked to HRP, as well as chemiluminescent reagents, were bought from Santa Cruz Biotech (Dallas, TX, USA).

Initially, all chemicals were dispersed in dimethylsulfoxide (DMSO) as a stock solution. The figure legends indicated the chemical treatment duration along with the final levels

### Cell death assessment and Western blot assays

After treatment with indicated chemicals, cells were supplemented with 10 µl of CCK-8 (BS350A; Biosharp, Hefei, China) solutions and 100 µl of medium each well. The OD values were determined at 450 nm after 1.5-h incubation. To verify the association of cell death with autophagy and ferroptosis, the classical autophagy markers (LC3, beclin-1, ATG12) and ferroptosis markers (ACSL4, FTH, NCOA4) were explored *via* Western blotting (15). We harvested the cells in a cold PBS solution after treatment, extracted the protein lysates, and subsequently performed immunoprecipitation with the specified antibodies in the figure.

### Malondialdehyde and glutathione assays

Cells were seeded in a six-well cell culture plate as indicated. The cell lysis was then collected and centrifuged. For the malondialdehyde (MDA) assay, the procedure was performed according to the instruction (Elabscience, Wuhan, China). For the GSH assay, the supernatant was collected and detected according to the specification of the GSH assay kit (Elabscience, Wuhan, China).

### Tumor immune estimation resource

TIMER2.0 constitutes a comprehensive web tool (http://timer.cistrome.org/) employed to analyze immune infiltrates in numerous kinds of cancers. We utilized the “Exploration-Gene DE” module to assess ACO1 along with IREB2 expressions in diverse malignancies. TIMER2.0 was adopted to explore the relationship of ACO1 and IREB2 with immune cell invasion in the KIRC. Using The Cancer Genome Atlas (TCGA) data resource, the “Immune-Gene” module can explore the relationship of ACO1 and IREB2 contents with immune cell invasion levels (B cell, dendritic cells, CD8+ T cell, neutrophils, CD4+ T cell, and neutrophils, as well as macrophages). We adopted the “Exploration-Gene Corr” module in TIMER2.0 to explore the link between ACO1 and IREB2 contents with numerous gene biomarker sets of immune cells. Purity-adjusted partial Spearman’s correlation along with the significance level was utilized to assess the relationships of ACO1 and IREB2 contents with immune invasion **(** **Supplemental Figure S3** **)**.

### The University of Alabama at Birmingham cancer data analysis portal (UALCAN)

UALCAN (http://ualcan.path.uab.edu/analysis.html) is a web-based resource offering in-depth assessments of transcriptome data from TCGA, MET500, and Clinical Proteomic Tumor Analysis Consortium data. UALCAN was used to explore the mRNA along with the protein contents of ACO1 and IREB2 genes in KIRC.

### The Human protein atlas database

The Human Protein Atlas (HPA) data resource (http://www.proteinatlas.org/) harbors extensive proteome along with transcriptome data for diverse human samples consisting of cell, tissue, and pathology atlases. The protein immunohistochemistry of IRP1 and IRP2 in normal kidney and renal cancer was obtained from this online website.

### XIANTAO platform

The XIANTAO platform (www.xiantao.love) is an online platform that contains TCGA data resource. The XIANTAO platform was used to analyze the expression of ACO1 and IREB2 and the relationship of ACO1, IREB2 with numerous clinicopathological indices (sex, histologic grades, age, cancer stages, nodal metastasis status along with distant metastasis status) of TCGA-KIRC. The patient samples were stratified into two groups on the basis of the median expression (high-ACO1 and IREB2 expression and low-ACO1 and IREB2 expression) to assess the OS (overall survival) and DSS (disease-specific survival) along with PFI (progress-free interval) with HR) with 95% Cis, as well as log-rank P-values. We chose the XIANTAO platform for assessing the forecast-worth of ACO1 and IREB2 in KIRC based on diversity clinicopathological parameters **(** **Supplemental Figures S1**, **2** **)**. Then, we created a concise nomogram and predicted the OS of KIRC in this platform, including nine factors. In addition, calibration curves were used for evaluating the predictive accuracy of the nomogram.

### Genemania and string databases

The GeneMANIA data resource (http://genemania.org/) was applied to construct the ACO1 and IREB2 crosstalk network. We adopted the STRING data resource (https://string-db.org/cgi/input) to create a protein–protein interaction (PPI) network of ACO1 and IREB2. Additionally, the relationship of ACO1 and IREB2 with iron metabolism-linked genes in KIRC were explored *via* Spearman’s correlation coefficient on the XIANTAO platform (www.xiantao.love).

### Gene expression profiling interactive analysis 2

GEPIA2 (http://gepia2.cancer-pku.cn/#index) a user-friendly web resource for analyzing gene expression of the TCGA along with GTEx data resources. In the module “Similar Genes Detection,” the coexpression genes of ACO1 and IREB2 were evaluated using TCGA-KIRC data sets. The top 300 coexpression genes with the Pearson correlation coefficient above 0.70 were utilized for GO (Gene Ontology) term and KEGG (Kyoto Encyclopedia of Genes and Genomes) pathway enrichment analysis on the XIANTAO platform (www.xiantao.love). GO analysis constitutes a bioinformatics tool to explore the BPs (biological processes), CCs (cellular components), and MFs (molecular functions) linked to ACO1 and IREB2. Then, in order to identify the potential pathways referring to ACO1 and IREB2 in KIRC, the KEGG pathway enrichment analysis was applied. Furthermore, the relationships of ACO1 and IREB2 with PD-1, LAG3, PD-L1, HAVCR2, and CTLA-4 were explored *via* Spearman’s correlation coefficient in the “Correlation Analysis” module.

### Human autophagy database

HADb (http://www.autophagy.lu/index.html) is a public data resource harboring data on the so- far-described human genes as involved in autophagy. A total of 222 genes were abstracted from the HADb. The differentially expressed autophagy-linked genes in KIRC were picked up and used for further analyses combined with ACO1 and IREB2.

### Ferroptosis database

FerrDb (http://www.zhounan.org/ferrdb/index.html) constitutes a data resource of ferroptosis modulators along with biomarkers, as well as ferroptosis–disease relationships. We abstracted 259 genes from the FerrDb. The differentially expressed ferroptosis-linked genes in KIRC were picked up for further analyses combined with ACO1 and IREB2.

These differential autophagy-associated genes and ferroptosis-associated genes in KIRC were conducted, respectively, for further analyses on the XIANTAO platform, such as correlation analysis, the PPI network, and GO along with KEGG pathway assessments.

### Kaplan–Meier plotter database analysis

The KM Plotter data resource (https://kmplot.com/analysis/) constitutes an online data resource harboring gene expression data and survival information coupled with the immune cell infiltration of 530 clinical KIRC patients.

This data resource was used to assess the prognostic significance of ACO1 and IREB2 in KIRC patients. On the basis of the mean expression, we stratified the samples into high-ACO1, -IREB2 expression and low ACO1, -IREB2 expression to determine the OS and DSS along with PFI, with the HRs of 95% CIs coupled with log-rank p-values **(** **Supplemental Figures S1**, **2** **)**. And then, this data resource was used to conduct a prognosis assessment on the basis of the contents of ACO1 or IREB2 in KIRC in linked immune cell subgroups. The OS was presented with HRs with 95% Cis along with log-rank p-values **(** **Supplemental Figures S4**, **5** **)**.

### Tumor immune infiltration analysis

The tumor immune invasion patterns were explored on the XIANTAO resource. To differentiate diverse immunocytes, we utilized a total of 24 immunological markers (28). The single-sample GSEA (ssGSEA) method was applied to compute the Spearman correlations of immunocyte biomarkers with ACO1 along with IREB2 expression levels (29).

### Tumor and immune system interaction database (TISIDB)

TISIDB (http://cis.hku.hk/TISIDB/index.php) constitutes an integrated data resource for tumor–immune system crosstalks. The links of ACO1, IREB2 with PD-1, LAG3, PD-L1, and CTLA-4, as well as HAVCR2, were explored *via* Spearman’s correlation coefficient in the “Immunomodulator” module.

### cBioPortal

The cBioPortal for Cancer Genomics supports visualization and abstraction, along with the assessment of the large-scale cancer genomics data set. We adopted the cBioPortal to further explore the TCGA-KIRC data sets (Firehose Legacy) with 538 cases. The “Plots” module was adopted to determine the changes in the copy numbers of ACO1 along with IREB2 in KIRC. On the XIANTAO platform (www.xiantao.love), the OS of ACO1, as well as IREB2 in CNA, was explored.

### Data presentation and statistical analysis

Every cell-based experiment was done in triplicate. The mean + SEM was employed to display quantitative data acquired from bioinformatics platforms (SEM). The Western blotting photos were representative of several blots. The bioinformatics platforms were used to create the graphic images. The analysis of variance (ANOVA) method was used for multiple group data analysis, followed by Student’s t-test or Wilcoxon rank-sum test for comparison between two groups. GraphPad Prism (version 7.0.0, San Diego, CA, USA) was used for statistical calculations, with P < 0.05 signifying statistical significance.

## Results

### ACO1 and IREB2 expression is decreased in kidney renal clear cell carcinoma patients

**Figure 1:**
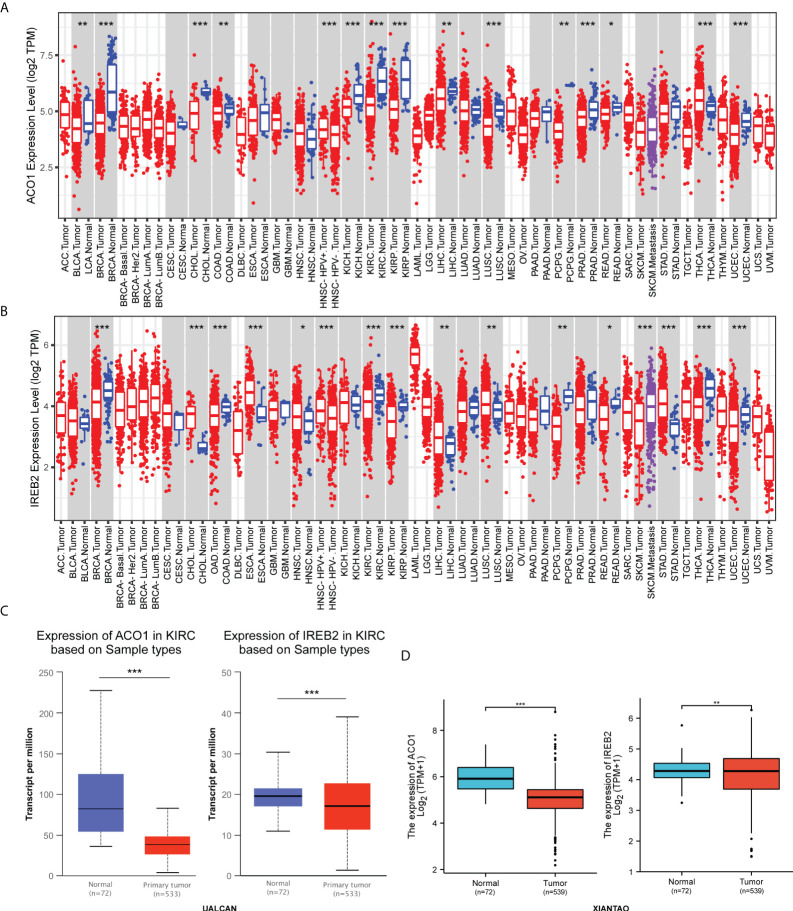
Expression of ACO1 and IREB2 in kidney renal clear cell carcinoma (KIRC). **(A, B)** ACO1 **(A)** and IREB2 **(B)** expression in different types of cancer was investigated with the Tumor Immune Estimation Resource (TIMER) database. **(C)** ACO1 and IREB2 expression in KIRC was examined by using the UALCAN database. **(D)** Analysis of ACO1 and IREB2 expression in KIRC and adjacent normal tissues in the TCGA database. *P < 0.05; **P < 0.01; ***P < 0.001.

The transcript contents of ACO1 and IREB2 in human solid cancers was first evaluated *via* the TIMER2.0 data resource. Both ACO1 and IREB2 genes were lowly expressed in breast-invasive carcinoma (BRCA), rectum adenocarcinoma (READ), cholangiocarcinoma (CHOL), colon adenocarcinoma (COAD), KIRC, pheochromocytoma, and paraganglioma (PCPG), as well as uterine corpus endometrial carcinoma (UCEC) assessed with the matching non-tumorous tissues **(** **Figures 1A, B** **)**. Concurrently, we also noticed that lower transcript contents of ACO1 and IREB2 was detected in KIRC tissues versus healthy kidney tissues in the UALCAN data resource **(** **Figure 1C** **)**. In addition, ACO1 and IREB2 contents in KIRC samples and neighboring tissues were assessed in data directly abstracted from the TGCA data resource. ACO1 and IREB2 contents were noticeably decreased in KIRC tissues **(** **Figure 1D** **)**. These findings illustrate that ACO1 and IREB2 expression are reduced in KIRC and indicate that ACO1 and IREB2 may harbor an essential modulatory role in KIRC advancement.

**Figure 2:**
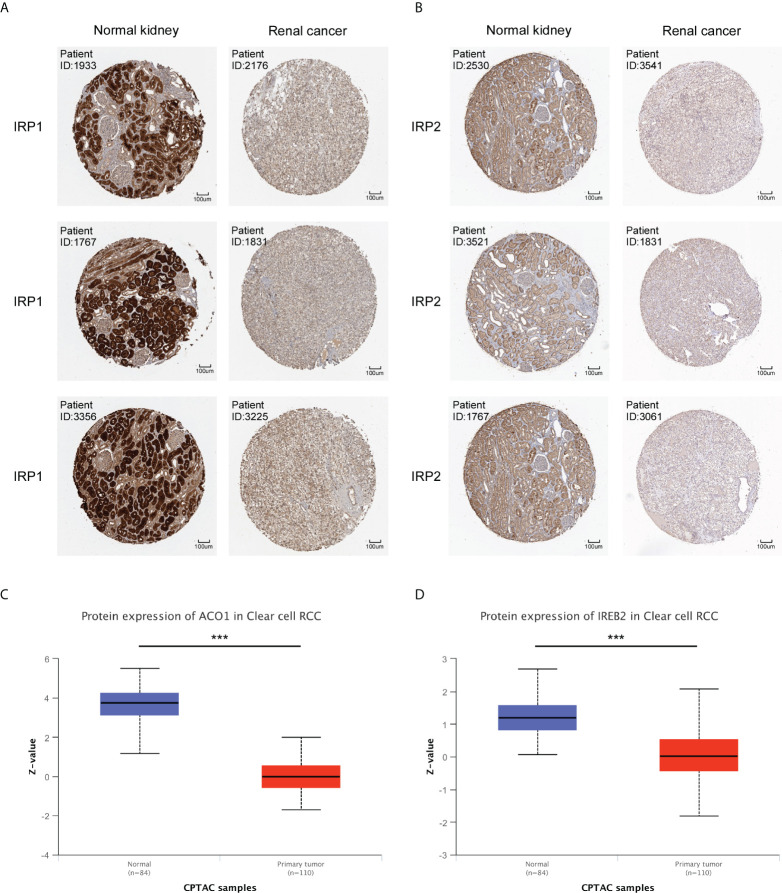
Protein expression of ACO1 and IREB2 in KIRC. **(A, B)** The protein expression levels of ACO1 **(A)** and IREB2 **(B)** by immunohistochemical staining from the HPA database (anti-ACO1 HPA024157, anti-IREB2 CAB032885, 10×). **(C, D)** ACO1 and IREB2 **(D)** protein expression in KIRC was analyzed by using the UALCAN database. ***P < 0.001.

The protein expression level of ACO1 and IREB2 were further investigated in renal cancer using the HPA database, and we noticed that the ACO1 and IREB2 protein level turned evidently down in the tissues of KIRC contrasted with healthy kidney **(** **Figures 2A, B** **)**. Consistently, the expression of ACO1 and IREB2 protein were evidently downregulated in KIRC tissue compared with healthy tissue in the UALCAN database **(** **Figures 2C, D** **)**.

### ACO1 and IREB2 expression and clinical parameters of the cancer genome atlas–kidney renal clear cell carcinoma patients

**Figure 3:**
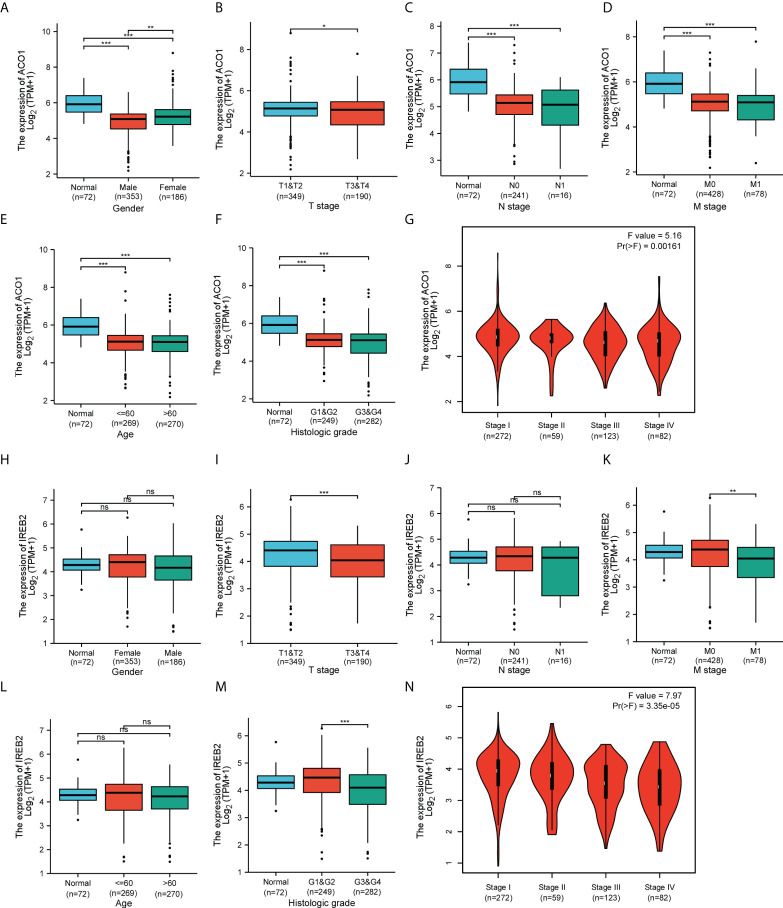
Box plots and violin plots valuating ACO1 and IREB2 expression among different groups of patients based on clinical parameters using the TCGA database. Analyses of ACO1 and IREB2 in KIRC were shown for sex **(A, H)**, TNM stage **(B–D, I–K)**, age **(E, L)**, histologic grade **(F, M)**, and cancer stage **(G, N)**. TNM: T: tumor, N: node, M: metastasis. Kruskal–Wallis Test and Dunn’s Test **(A, C–F, H, J–M)**; Wilcoxon rank sum test **(B, I)**; one-way ANOVA **(G, N)**. ns (not significant); *P < 0.05; **P < 0.01; ***P < 0.001.

By applying the online tool of the XIANTAO platform, we then explored ACO1 and IREB2 expression among different groups of patients divided by clinical parameters **(** **Supplemental Table 1** **)**. In line with sex, ACO1 expression was noticeably downregulated in TCGA-KIRC samples among both men and women when contrasted to the matching normal controls **(** **Figure 3A** **),** but not IREB2 **(** **Figure 3H** **).** Based on the TNM stage, ACO1 and IREB2 expression were significantly decreased in KIRC patients with T3 and 4 relative to those with T1 and 2 **(** **Figures 3B, I** **)**. ACO1 expression, but not IREB2 expression, was lower in KIRC patients that are classified as N0 or N1 compared with normal controls **(** **Figures 3C, J** **)**. A noticeable decrease in ACO1 expression was watched among KIRC patients classified as M0 or M1 contrasted with normal controls, while a remarkable drop in the IREB2 level was witnessed in KIRC patients with M1 relative to those with M0 **(** **Figures 3D, K** **)**. In terms of age, ACO1 expression, but not IREB2 expression, was remarkably reduced in the KIRC tissues of patients from different age groups compared with normal controls **(** **Figures 3E, L** **)**. In addition, ACO1 expression was dramatically decreased in KIRC patients in grade 1, 2, 3, and 4 compared with normal controls, while IREB2 expression was significantly reduced in KIRC patients in grade 3 and 4 compared with KIRC patients in grade 1 and 2 (**Figures 3F, M**). Regarding the tumor stage, a remarkable decrease in ACO1 and IREB2 expression was watched in KIRC patients in stages I, II, III, and IV **(** **Figures 3G, N** **)**.

### Decreased ACO1 and IREB2 expression correlates with poor prognosis in the cancer genome atlas–kidney renal clear cell carcinoma patients

**Figure 4:**
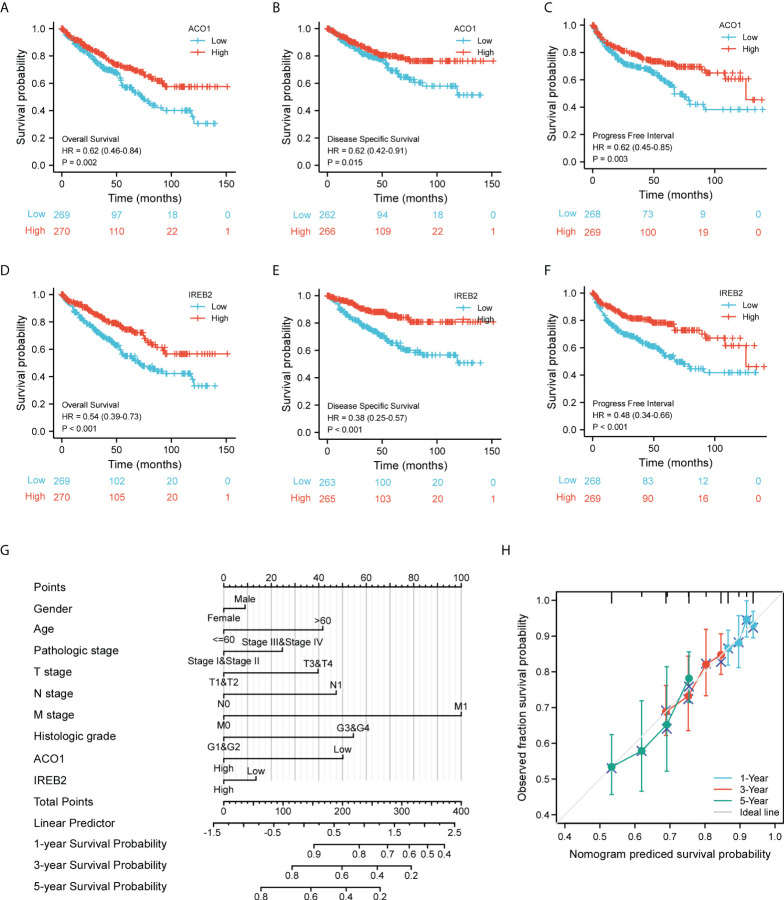
Evaluation for the prognostic value of ACO1 and IREB2 in KIRC patients of TCGA cohort. **(A–C)** Survival curves of ACO1 were shown for overall survival (OS) **(A)**, disease-specific survival (DSS) **(B)**, and progress-free interval **(C)**. **(D–F)** Survival curves of IREB2 were shown for OS **(D)**, DSS, **(E)** and progress-free interval **(F)**. **(G)** Nomogram for predicting the probability of 1-, 3- and 5-year OS for KIRC patients of the TCGA cohort. **(H)** Calibration curves for nomogram predictions. The calibration curves for predicting 1-, 3-, and 5-year OS for KIRC patients of the TCGA cohort. TCGA, The Cancer Genome Atlas; KIRC, kidney renal clear cell carcinoma.

Considering the expression level of ACO1 and IREB2 were closely related to progression and metastasis in KIRC patients, we then assessed their value in cancer prognosis **(** **Tables 1**, **2** **)**. We explored the databases from TCGA by using the XIANTAO platform and then found that KIRC patients with a lower expression of ACO1 or IREB2 gene exhibited worse OS, DSS, and PFI **(** **Figures 4A–F** **)**. These results indicate that ACO1 and IREB2 are strongly linked with the predicting outcomes of KIRC patients.

**Table 1:** ACO1 single-gene logistic regression.

Characteristics	Total (N)	Odds Ratio (OR)	P-value
Age (>60 vs. ≤60)	539	0.935 (0.667-1.311)	0.698
Gender (Male vs. Female)	539	0.655 (0.457-0.936)	0.020
Pathologic stage (Stage III and Stage IV vs. Stage I and Stage II)	536	0.708 (0.499-1.004)	0.053
T stage (T3 and T4 vs. T1 and T2)	539	0.792 (0.555-1.128)	0.196
N stage (N1 vs. N0)	257	0.687 (0.238-1.901)	0.470
M stage (M1 vs. M0)	506	0.877 (0.540-1.423)	0.596
Histologic grade (G3 and G4 vs. G1 and G2)	531	0.961 (0.683-1.351)	0.817
Primary therapy outcome (PR and CR vs. PD and SD)	147	1.312 (0.473-3.700)	0.599
Serum calcium (Normal vs. Elevated and Low)	366	1.095 (0.722-1.660)	0.670
Hemoglobin (Normal vs. Elevated and Low)	459	1.413 (0.975-2.054)	0.069
Laterality (Right vs. Left)	538	0.984 (0.701-1.381)	0.927
Race (White vs. Asian and Black or African American)	532	0.974 (0.578-1.639)	0.920

**Table 2:** IREB2 single-gene logistics regression.

Characteristics	Total (N)	Odds Ratio (OR)	P-value
Age (>60 vs. ≤60)	539	0.716 (0.509-1.004)	0.053
Gender (Male vs. Female)	539	0.592 (0.413-0.847)	0.004
Pathologic stage (Stage III and Stage IV vs. Stage I and Stage II)	536	0.450 (0.314-0.642)	<0.001
T stage (T3 and T4 vs. T1 and T2)	539	0.498 (0.346-0.712)	<0.001
N stage (N1 vs. N0)	257	0.913 (0.326-2.557)	0.860
M stage (M1 vs. M0)	506	0.456 (0.272-0.748)	0.002
Histologic grade (G3 and G4 vs. G1 and G2)	531	0.540 (0.382-0.762)	<0.001
Primary therapy outcome (PR and CR vs. PD and SD)	147	1.397 (0.504-3.939)	0.518
Serum calcium (Normal vs. Elevated and Low)	366	0.714 (0.468-1.085)	0.116
Hemoglobin (Normal vs. Elevated and Low)	459	1.578 (1.087-2.297)	0.017
Laterality (Right vs. Left)	538	1.213 (0.865-1.704)	0.264
Race (White vs. Asian and Black or African American)	532	1.027 (0.610-1.730)	0.920

We develop a nomogram that illustrate the ACO1- and IREB2-related danger signature and several independent clinical indexes (gender, age, pathologic stage, TNM stage, and histologic stage), which may provide doctors a quantitative method to assess the predicting outcomes of KIRC patients **(** **Figure 4G** **)**. We also performed Cox univariate and multivariate analyses to verify the independent roles of these risk factors (age, gender, sex, TNM stage, pathologic stage, and histologic stage) including ACO1 and IREB2 **(** **Supplemental Table 2** **)**. Furthermore, a favorable consistency among the prediction and actual study was illustrated by the calibration plot of the nomogram in the first-year, third-year, and fifth-year OS in the TCGA-KIRC cohort **(** **Figure 4H** **).**

### Association of ACO1 and IREB2 expression with various clinicopathological features in the cancer genome atlas–kidney renal clear cell carcinoma patients

To further study the predictive value and possible mechanism of ACO1 and IREB2 differential expression in KIRC, we used the Kaplan–Meier database to seek the potential links between ACO1 and IREB2 mRNA expression and clinical features. Low ACO1 expression was remarkably accompanied with worse OS, DSS, and PFI only in the female cohort in KIRC **(** **Supplemental Figure S1** **)**, while low IREB2 expression was noticeably accompanied with unsatisfied OS, DSS, and PFI in KIRC patients **(** **Supplemental Figure S2** **)**. In different tumor stages, low ACO1 expression was accompanied with worse OS, DSS, and PFI only in stage 1 and 3 patients with KIRC **(** **Supplemental Figure S1** **)**, while low IREB2 expression was strongly accompanied with worse OS in stage 1,worse DSS in stage 3, and worse PFI in stage 1 and 3 patients with KIRC **(** **Supplemental Figure S2** **)**. Based on the histologic grade, low ACO1 and IREB2 expression were remarkably accompanied with worse OS, DSS, and PFI in grade 2 and 3 patients with KIRC **(** **Supplemental Figures S1**, **2** **)**.

In terms of the TNM stage, a noticeable link between low ACO1 expression and worse OS, DSS, and PFI was studied in American Joint Committee on Cancer (AJCC) stage T-1 and T-3 KIRC patients **(** **Supplemental Figure S1** **)**, while a significant clue between low IREB2 expression and worse OS, DSS, and PFI were detected in AJCC stage T-1 and T-3 KIRC patients **(** **Supplemental Figure S2** **)**. Low ACO1 expression was noticeably accompanied with unsatisfied OS and DSS in N0 KIRC patients **(** **Supplemental Figure S1** **)**, while low IREB2 expression was noticeably accompanied with worse OS, DSS, and PFI in N0 patients with KIRC **(** **Supplemental Figure S2** **)**. Low ACO1 and IREB2 expression was remarkably accompanied with worse OS, DSS, and PFI in M0 patients with KIRC **(** **Supplemental Figures S1**, **2** **)**.

In addition, a significant clue between low ACO1 expression and worse OS, DSS, and PFI were observed in ectopic serum calcium patients with KIRC **(** **Supplemental Figure S1** **)**, while a significant link between poor IREB2 expression and worse OS, DSS, and PFI was observed in both ectopic and normal serum calcium patients with KIRC **(** **Supplemental Figure S2** **)**. Furthermore, we noticed a strong link between poor ACO1 and IREB2 expression and worse OS, DSS, and PFI in both ectopic and normal hemoglobin patients with KIRC **(** **Supplemental Figures S1**, **2** **)**. Furthermore, low ACO1 and IREB2 expression was strongly accompanied with unsatisfied OS, DSS, and PFS in the cohort with left or right laterality KIRC **(** **Supplemental Figures S1**, **2** **)**. These phenomena imply that ACO1 and IREB2 mRNA expression could show a remarkable forecast-value in patients with KIRC.

### Identification of ACO1- and IREB2-interacting genes and proteins

**Figure 5:**
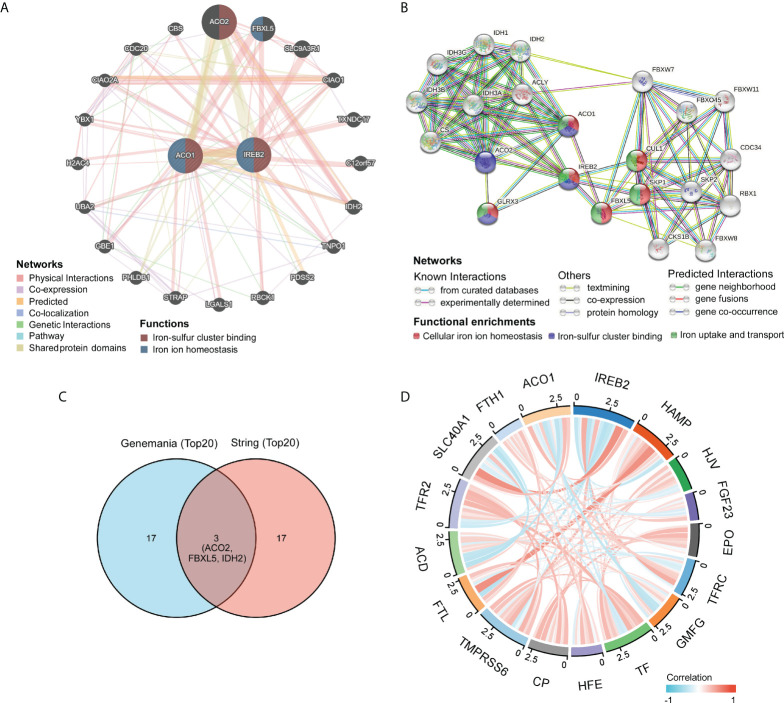
Identification of ACO1- and IREB2-interacting genes and proteins. **(A)** The gene–gene interaction network of ACO1 and IREB2 was constructed using GeneMania. **(B)** The protein–protein interaction (PPI) network of ACO1 and IREB2 was generated using STRING. **(C)** A Venn diagram showing the overlap of top 20 genes and proteins predicted by GeneMania and STRING. **(D)** A chord diagram shows the correlations between ACO1, IREB2 and iron metabolism–related genes in KIRC.

The gene–gene crosstalk network for ACO1, IREB2, and the modified adjacent genes was constructed *via* GeneMania. It reminded that the 20 most changeable genes were remarkably accompanied with ACO1 and IREB2, consisting of SLC9A3R1, FBXL5, and ACO2 **(** **Figure 5A** **)**. Further functional analysis revealed that these genes were remarkably accompanied with the iron–sulfur cluster binding and iron ion homeostasis **(** **Figure 5A** **)**. We created a PPI network of ACO1 and IREB2 with the STRING data resource **(** **Figure 5B** **)**, which showed 176 edges and 22 nodes, consisting of GLRX3 and CUL1 along with SKP1 **(** **Figure 5B** **)**. It was suggested in functional enrichments that these genes were noticeably accompanied with the cellular iron ion homeostasis, iron-sulfur cluster binding, and iron uptake and transport **(** **Figure 5B** **)**. The overlap of top 20 genes and top 20 proteins predicted by GeneMania and STRING were ACO2, FBXL5, and IDH2 **(** **Figure 5C** **)**. Furthermore, we used the TCGA data resource to investigate the relationship of ACO1 and IREB2 with iron metabolism-linked genes; and then, we found that ACO1 was directly, as well as noticeably, linked to IREB2, TFRC, HFE, SLC40A1, and FTH1 but inversely associated with HAMP, HJV, FGF23, GMFG, TF, TMPRSS6, ACD, and TFR2 in KIRC **(** **Figure 5D** **)**. IREB2 was directly, as well as noticeably, linked to ACO1, TFRC, HFE, and SLC40A1; however, IREB2 was inversely associated with HAMP, HJV, GMFG, TF, TMPRSS6, FTL, ACD, and TFR2 in KIRC **(** **Figure 5D** **)**. These results remind that ACO1 and IREB2 are associated with the modulation of iron uptake, transport, and cellular iron ion homeostasis.

### Gene ontology (GO) and kyoto encyclopedia of genes and genomes (KEGG) pathway analysis of ACO1, IREB2 and its coexpressed genes in TCGA-KIRC

**Figure 6:**
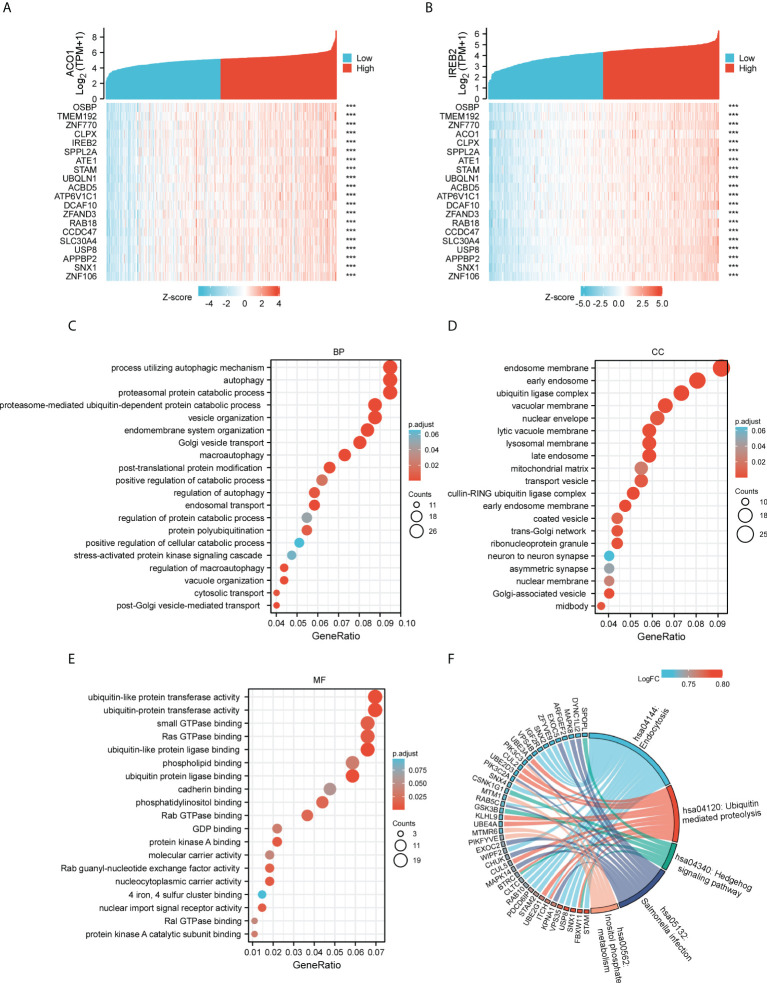
Gene Ontology (GO) and Kyoto Encyclopedia of Genes and Genomes (KEGG) enrichment analysis for ACO1 and IREB2 in TCGA-KIRC database. **(A, B)** Heat maps showing the top 20 coexpression genes positively correlated with ACO1 **(A)** and IREB2 **(B)** in KIRC. **(C–E)** Top 20 enrichment terms in BP **(C)**, CC **(D)**, and MF **(D)** categories in KIRC. **(F)** A chord diagram shows KEGG enrichment pathways in KIRC. GO: Gene Ontology; KEGG: Kyoto Encyclopedia of Genes and Genomes; TCGA, The Cancer Genome Atlas; KIRC, kidney renal clear cell carcinoma; BP, biological process; CC, cellular component; MF, molecular function.

Our team determined genes directly or inversely coexpressed with ACO1 and IREB2 by mining data from the GEPIA2 data resource. The 20 most plentiful genes that coexpressed among ACO1 and IREB2 in KIRC are shown **(** **Figures 6A, B** **)**. Then, we used KEGG and GO enrichment analyses for 300 genes coexpressed with ACO1 and IREB2 to explore the ACO1- and IREB2-linked cascades along with biological functions. The top 20 essential terms of BP, MF, and CC enrichment analyses are listed **(** **Figures 6C–E** **)**. The KEGG cascades for ACO1, IREB2 and its linked genes are illustrated in **Figure 6F**. Notably, in terms of BP, ACO1 and IREB2 were enriched in autophagy-linked processes, such as autophagy, macroautophagy, the modulation of autophagy, a process utilizing autophagic mechanism, and the modulation of macroautophagy **(** **Figure 6C** **)**. These results strongly imply that ACO1 and IREB2 participated in the modulation of autophagy in KIRC patients.

### Correlation analysis of ACO1 and IREB2 expression with autophagy-related genes in the cancer genome atlas–kidney renal clear cell carcinoma database

**Figure 7:**
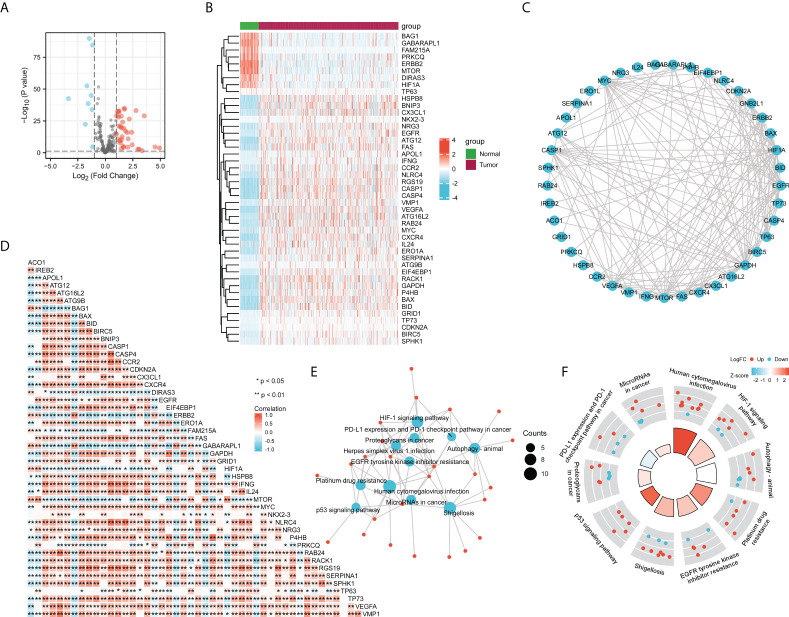
Correlation analysis of ACO1, REB2 expression with autophagy-related genes in TCGA-KIRC database. **(A, B)** Volcano plots **(A)** and heat maps **(B)** showing the 45 differentially expressed autophagy-related genes in TCGA-KIRC database compared with normal controls. **(C)** A heat map shows the correlations between ACO1, IREB2 and the 45 genes in KIRC. **(D)** The PPI network of ACO1, IREB2 and the 45 genes was generated using STRING. **(E)** The visualized network of KEGG enrichment analysis for ACO1, IREB2 and the 45 genes. **(F)** A circos diagram shows KEGG enrichment pathways with the z-score for ACO1, IREB2 and the 45 genes. PPI, protein–protein interaction.

To further evaluate the correlation of ACO1 and IREB2 expression with autophagy, a total of 222 autophagy-related genes were obtained from the Human Autophagy Database (HADb). Compared with the normal controls, we identified the 45 differentially stated autophagy-related genes in TCGA-KIRC by the criteria of P-value < 0.05 and absolute logarithmic (base 2) fold-change value >1.0 **(** **Figures 7A, B** **)**. The 45 differentially stated autophagy-related genes, including 36 upmodulated and 9 downmodulated genes, were presented in volcano plots and heat maps **(** **Figures 7A, B** **)**.

To figure the interactions among ACO1, IREB2 and the 45 differentially stated autophagy-related genes, we performed PPI analysis. The results illustrated that ACO1 and IREB2 were cointeracted with HIF1A and GAPDH **(** **Figure 7C** **)**. To further explore the expression correlation of ACO1, IREB2 with the 45 genes in KIRC, correlation analysis was performed built on TCGA database. ACO1 was noticeably correlated with 38 of 45 autophagy genes in KIRC, and IREB2 was significantly correlated with 37 of 45 autophagy genes in KIRC **(** **Figure 7D** **)**.

We choose GO along with KEGG enrichment analysis in the XIANTAO platform to scrutinize the possible biological roles of these variously expressed autophagy-associated genes combined with ACO1 and IREB2. The data demonstrated that the essential GO terms participated in the process utilizing autophagic mechanism, autophagy, and macroautophagy (biological process); autophagosome and autophagosome membrane (cellular component); and ubiquitin-like protein ligase binding and ubiquitin protein ligase docking (molecular function) **(** **Supplemental Figures S3A–C** **)**. In KEGG enrichment analysis, the ACO1, IREB2 and 45 differentially expressed autophagy-linked genes mainly participated in the procedure of autophagy **(** **Figures 7E, F** **)**. In addition, ACO1 and IREB2 were predicted to correlate with the p53 signaling cascade and PD-L1 level and PD-1 checkpoint cascade in solid cancer **(** **Figures 7E, F** **)**, which supports the relationship of ACO1, IREB2 with immune invasion in KIRC.

### Correlation analysis of ACO1 and IREB2 expression with ferroptosis-related genes in the cancer genome atlas–kidney renal clear cell carcinoma database

**Figure 8:**
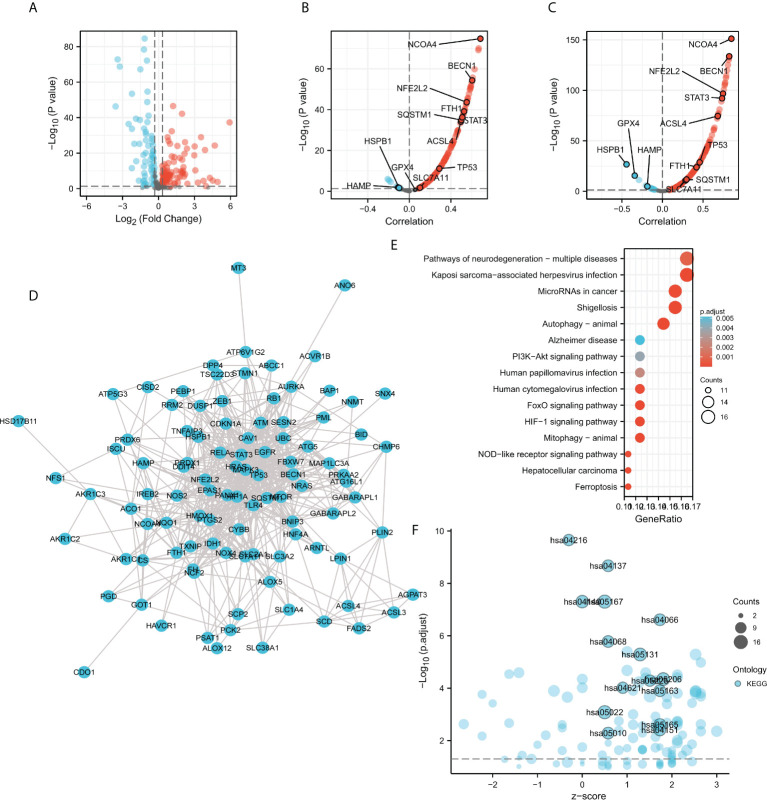
Correlation analysis of ACO1, REB2 expression with ferroptosis-related genes in TCGA-KIRC database. **(A)** Volcano plots showing the 150 differentially expressed ferroptosis-related genes in the TCGA-KIRC database compared with normal controls. **(B, C)** Volcano plots show the correlations between ACO1 **(B)**, IREB2 **(C)**, and the 150 ferroptosis-related genes in KIRC. **(D)** The PPI network of the 150 genes was generated using STRING. **(E)** A bubble chart shows top 15 KEGG enrichment pathways for the 150 genes in KIRC. **(F)** A bubble chart shows top 15 KEGG enrichment pathways with z-score for the 150 genes.

Given the potential association with cellular iron ion homeostasis **(** **Figure 5D** **)** and autophagy **(** **Figures 6C**, **7E** **)**, the correlations of ACO1 and IREB2 with ferroptosis were further evaluated in the TCGA-KIRC database. A total of 259 ferroptosis-related genes were learned from the Ferroptosis Database (FerrDb). Compared with the normal controls, the 150 ferroptosis-associated genes expressed distinctively, including ACO1, in TCGA-KIRC were distinguished using the criteria of P-value <0.05 and absolute logarithmic (base 2) fold-change value >0.325 **(** **Figure 8A** **)**. The 150 differentially expressed ferroptosis-related genes, along with 87 upmodulated genes and 63 downmodulated genes, are illustrated in volcano plots **(** **Figure 8A** **)**.

To further explore the expression correlation of ACO1, IREB2 with the 150 genes in KIRC, correlation analysis was performed based on the TCGA database. ACO1 was noticeably accompanied with 121 of 150 ferroptosis-related genes in KIRC, and IREB2 was significantly correlated with 130 of 150 ferroptosis-related genes in KIRC **(** **Figures 8B, C** **)**. A total of 111 genes were correlated with both ACO1 and IREB2. To further determine the interactions among ACO1, IREB2 and the 111 ferroptosis-related genes, PPI analysis was performed. The results demonstrated that ACO1 and IREB2 were cointeracted with NFS1, FTH1, HAMP, FH, CS, ISCU, EPAS1, NCOA4, HIF1A, GOT1, and HMOX1 **(** **Figure 8D** **)**.

The XIANTAO platform was used and GO and KEGG enrichment analyses were conducted to evaluate the possible biological functions of the 111 genes accompanied with ACO1 and IREB2. The outcomes suggested that the essential GO-enriched terms participated in procedure response to oxidative stress, autophagy, and response to metal ion (biological process); mitochondrial outer membrane, autophagosome, and vacuolar membrane (cellular component); and ubiquitin protein ligase binding, iron ion binding, and antioxidant activity (molecular function) **(** **Supplemental Figures S4A–C** **)**. In KEGG enrichment analysis, the ACO1, IREB2 and the 111 genes were chiefly involved in the process of autophagy-animal, mitophagy-animal, the HIF-1 signaling pathway, and ferroptosis **(** **Figures 8E, F** **)**. These results suggested that ACO1 and IREB2 were tightly associated with autophagy and ferroptosis in KIRC.

### Relationship of ACO1 and IREB2 contents with invading immune cells

**Figure 9:**
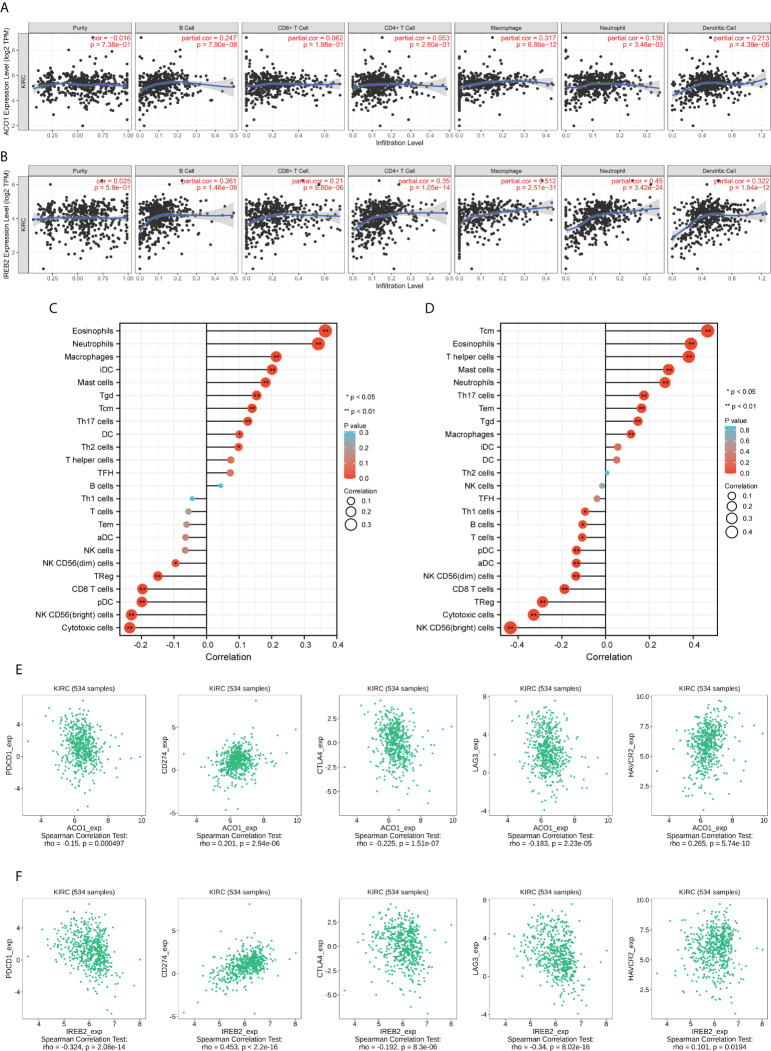
ACO1 and IREB2 expression positively correlates with immune infiltration. The correlation of ACO1 and IREB2 expression levels with the immune infiltration in KIRC tissues was analyzed using TCGA dataset. **(A, B)** Correlation analyses of ACO1 **(A)** and IREB2 **(B)** with the infiltration of different immune cells using the TIMER2.0 database. **(C, D)** A lollipop chart showed the correlations of ACO1 **(A)**, IREB2 **(B)** for all 24 immune cell types. iDC, immature DC; Tem, effector memory T cells; TFH, follicular helper T cells; Tgd, gamma delta T cells; pDC, plasmacytoid DC; aDC, activated DC; Tcm, central memory T cells using the XIANTAO platform. **(E, F)** Scatter plots of the correlations between ACO1 **(E)** and IREB2 **(F)** expression and PD-1, PD-L1, CTLA-4, LAG3, and HAVCR2 in KIRC using the TISIDB dataset.

The links between ACO1 and IREB2 expression with six kinds of invading immune cells were explored, consisting of B cells, neutrophils, CD8+ T cells, macrophages, CD4+ T cells, and dendritic cells. The data announced that ACO1 contents had a remarkably direct association with the invasion of B cells, dendritic cells, and macrophages along with neutrophils, while there were no noticeable relationships with CD4+ T cells and CD8+ T cells in KIRC **(** **Figure 9A** **)**. Meanwhile, there were direct and remarkable links between the expression of IREB2 with the invasion of all six kinds of immune cells in KIRC **(** **Figure 9B** **)**.

We assessed the links between ACO1 and IREB2 and immune invasion by using the accomplished data resource CIBERSORT, which reflects the influences of ACO1 and IREB2 on the tumor microenvironment (TME). In KIRC, ACO1 was established to be directly linked to eosinophils, neutrophils, macrophages, immature dendritic cells (iDC), mast cells, gamma-delta T cells (Tgd), central memory T cells (Tcm), type 17 T helper cells (Th17 cells), and dendritic cells (DC), along with type 2 T helper cells (Th2 cells); however, ACO1 was inversely linked to cytotoxic cells, NK CD56(bright) cells, plasmacytoid dendritic cells (pDC), CD8 T cells, and regulatory T cells (TReg), as well as NK CD56(dim) cells **(** **Figure 9C** **)**. Moreover, in KIRC, IREB2 was directly linked to Tcm cells, eosinophils cells, T helper cells, mast cells, neutrophils, Th17 cells, effector memory T cells (Tem), and Tgd, along with macrophages; however, IREB2 was inversely linked to NK CD56 (bright) cells, cytotoxic cells, TReg, CD8 T cells, NK CD56(dim) cells, activated DC (aDC), pDC, T cells, and B cells, along with Th1 cells **(** **Figure 9D** **)**.

### Relationship of hepcidin contents with diverse immune markers

We introduced the TIMER data resource to verify the links between ACO1 and IREB2 expression and multiple immune signatures in KIRC, which deepen our comprehension of ACO1 and IREB2 crosstalk with the immune response. We used the genes listed in the **Supplemental Figure S5A** to characterize immune cells consisting of M2 macrophages, B cells, tumor-associating macrophages (TAMs), T cells, dendritic cells, CD8+ T cells, neutrophils, monocytes, and M1 macrophages, as well as natural killer (NK) cells. Tumor purity is a pivotal factor in determining how immune invasion is dissected in clinical cancer biopsies. After the purity of the tumor was adjusted, ACO1 along with IREB2 expressions was remarkably linked to most immune markers in various kinds of immune cells in KIRC **(** **Supplemental Figure S5A** **)**.

We also studied the potential link between ACO1 and IREB2 contents and diverse functional T cells, consisting of resistant memory T cells, Th1, effector Tregs, effector memory T cells, Th1-like, Treg, naïve T cells, resting Tregs, Th2, and effector T cells along with exhausted T cells **(** **Supplemental Figure S5B** **)**. When using the TIMER data resource, we noticed that the ACO1 and IREB2 contents were strongly accompanied with 33 of 38 T-cell biomarkers in KIRC after adjusting for tumor purity **(** **Supplemental Figure S5B** **)**.

Then, the interrelationship of ACO1 and IREB2 expression with famous T-cell checkpoints, consisting of PD-1, HAVCR2, PD-L1, LAG3, and CTLA-4, were developed in the GEPIA data resource. Both ACO1 and IREB2 expression were strongly accompanied with the contents of PD-1, LAG3, PD-L1, CTLA-4, and HAVCR2 in KIRC **(** **Figures 9E, F** **)**. These conclusions deeply support that ACO1 and IREB2 expression are strongly linked to immune infiltration and imply that ACO1 and IREB2 play important roles in immune escape in the KIRC microenvironment.

### Prognostic analysis of ACO1 and IREB2 expression based on immune cells in kidney renal clear cell carcinoma patients

**Figure 10:**
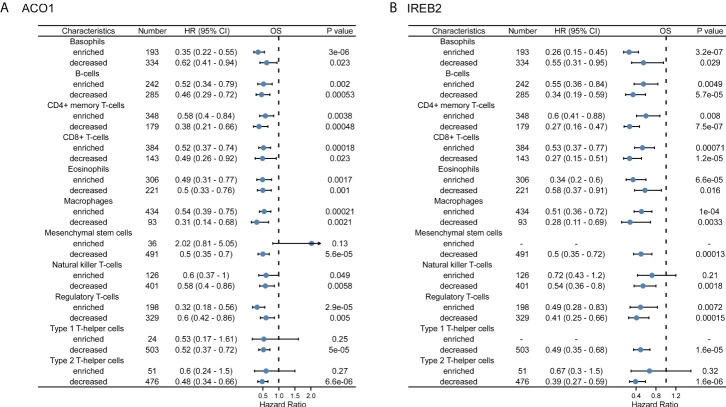
A forest plot shows the prognostic value of ACO1 **(A)** and IREB2 **(B)** expression according to different immune cell subgroups in KIRC patients.

Accordingly, ACO1 and IREB2 expression is closely accompanied with immune invasion and even worse prognosis in KIRC; we explored if the prognosis of KIRC because of immune invasion was modulated by ACO1 and IREB2 expression. We developed prognosis assessments built on the contents of ACO1 or IREB2 in KIRC in corresponding immune cell subgroups. As exhibited in **Figures 10A, B**, there were KIRC individuals with a low expression of ACO1 and poor invasion state of mesenchymal stem cells, type 1 T-helper cells. The type 2 T-helper cells harbored worse prognosis. Furthermore, there were remarkable correlations of ACO1 contents with the prognosis of KIRC in the cohort with different levels of B cells, regulatory T cells, CD4+ memory T cells, basophils, CD8+ T cells, eosinophils, and macrophages, as well as natural killer T-cells infiltrations **(** **Figure 10A** and **Supplemental Figure S6** **)**. Consistently, KIRC patients who express a low level of IREB2 and poor infiltration state of mesenchymal stem cells, type 1 T helper cells, natural killer T cells, and type 2 T helper cells had a worse prognosis. There were noticeable links between ACO1 contents and the prognosis of KIRC in the cohort with diverse levels of basophils, eosinophils, B-cells, CD4+ memory T-cells, regulatory T-cells, and CD8+ T-cells, along with macrophage infiltration **(** **Figure 10B**, **Supplemental Figure S7** **)**. These conclusions imply that the prognosis of KIRC patients may be affected by the immune infiltration of ACO1 and IREB2.

### ACO1 inhibitor, sodium oxalomalate, and IREB2 inhibitor, sodium nitroprusside, reduces sorafenib-triggered cell death in renal cancer cells

**Figure 11:**
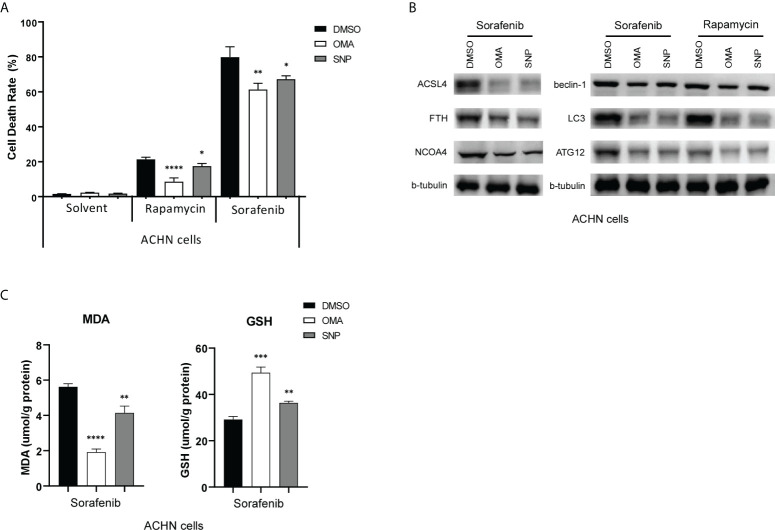
ACO1 or IREB2 inhibitor pretreatment reduces sorafenib-induced and rapamycin-induced cell death in renal cancer cells. **(A, B)** ACHN cells were pretreated with OMA (5 mM) for 24 h or with SNP (100 µM) for 6 h, followed by sorafenib (10 µM) or rapamycin (10 nM) treatment for 24 h. Cells were harvested for the Cell Counting Kit-8 assay **(A)** or Western blot assays with the antibodies as indicated **(B)**. b-tubulin blots served as the protein loading control. The asterisk indicates a significant difference compared to the DMSO control (P < 0.05, Student’s t-test). **(C)** Concentrations of MDA (left) and GSH (right) were detected with indicated treatments. OMA, sodium oxalomalate; SNP, sodium nitroprusside; DMSO, dimethylsulfoxide; MDA, malondialdehyde; GSH, glutathione. *P < 0.05; **P < 0.01; ***P < 0.001 ****P < 0.0001.

Sorafenib is a multikinase inhibitor, which is commonly used for treating advanced kidney and liver cancer. It has been shown to induce ferroptosis in renal cancer cells (32). A previous study reported that the knockdown of IREB2 with small interfering RNA (siRNA) readily blocked the cytotoxic effects of sorafenib in HCC cells (38). We then wondered if blocking ACO1 and IREB2 action interferes with sorafenib-triggered cell death in renal cancer. An ACO1 inhibitor, sodium oxalomalate (OMA) (39), and IREB2 inhibitor, sodium nitroprusside (SNP) (40), were employed to pretreat ACHN cells for 6 h, then treated with sorafenib for 24 h. As illustrated in **Figure 11A**, treating with sorafenib leads to a noticeable cell death-triggering effect on both renal cancer cells. Cell death induced by sorafenib moderately ameliorated after pretreatment with OMA or SNP. Sorafenib-triggered autophagy, as evaluated by LC3, BECLIN1, and ATG12, was strongly downregulated in both cell kinds preinoculated with OMA or SNP, and similar results were also obtained after treating with rapamycin **(** **Figures 11B** **)**. Previous reports in line with this conclusion that sorafenib-triggered cell death is an autophagy response (41). Furthermore, the inhibition of ACO1 or IREB2 in OMA or SNP preinoculated cells reduced ACSL4, FTH, and NCOA4 protein contents upon treatment with sorafenib **(** **Figures 11B** **)**. Consistently, the repression of ACO1 or IREB2 by OMA or SNP attenuated MDA, while it improved glutathione (GSH) levels in ACHN cells treated with sorafenib **(** **Figures 11C** **)**. These data imply that ACO1 and IREB2 participated in sorafenib-induced ferroptosis and blocking ACO1 or IREB2 could attenuate sorafenib’s anticancer influence. Alternatively, overexpressing or enhancing the enzyme activity of ACO1 and IREB2 might be novel treatment targets for sorafenib-resistant KIRC patients.

### Aberrant deletion of copy-number alterations are involved in ACO1 and IREB2 downexpression in KIRC and are correlated with poor prognostic outcomes

In the diversity of cancers, the ACO1 locus on chromosome 9p21.1 and IREB2 locus on chromosome 15q25.1 are generally deleted (42, 43). The CNA of ACO1 and IREB2 in TCGA-KIRC data sets are illustrated in **Supplemental Figures 8A, B**. The levels of ACO1 and IREB2 mRNA were closely correlated to the CNA **(** **Supplemental Figures S8C, D** **)**. The deletion in CNA of ACO1 and IREB2 predicted a lower gene expression, while the amplification in CNA of ACO1 and IREB2 predicted a higher gene expression **(** **Supplemental Figures S8E, F** **)**. Notably, KIRC patients with ACO1 or IREB2 deletion was found to be a predictor of poorer survival outcomes than those without deletion (P < 0.001, **Supplemental Figure S8G**; P = 0.003, **Supplemental Figure S8H**). These results indicated that aberrant ACO1 or IREB2 deletion play a role in their down-expression in KIRC, which conferred a poor prognosis.

## Discussion

RCC was reported as the second-most frequent urological malignancy, and kidney renal clear cell carcinoma (KIRC) takes place in 85% of these cases (1). Normally, surgical resection is chosen as the first line of treatment for RCC, while up to 40% of patients are accompanied with a local relapse and distant metastases (1). It is essential to explore potential mechanisms that are involved in the occurrence of RCC metastasis and develop powerful prognostic signatures of RCC. Herein, we evaluated the bioinformatics analyses of the TIMER2.0, UALCAN, TCGA, and HPA public data resources and noticed that the contents of ACO1 and IREB2 in KIRC were lower than healthy kidney tissue **(** **Figures 1**, **2** **)**. Previous reports were in line with this conclusion that ACO1 and IREB2 may work as an oncogene by pushing the occurrence and progress of RCC (34). Afterwards, we explored the clinical prognostic worth of ACO1 and IREB2 in KIRC patients. A low expression of ACO1 and IREB2 was noticeably associated with sex, histological grade, age, TNM stage, and clinical stage in KIRC patients **(** **Figure 3** **)**. Referring to the patient specimens in the cBioPortal data resource, there were nearly 28% of KIRC patients with ACO1 deletion in CNA, while there were approximately 6.9% of KIRC patients with IREB2 deletion in CNA. Aberrant ACO1 or IREB2 deletion contributes to the downexpression of ACO1 and IREB2 in KIRC, which confers poor prognostic outcomes. Furthermore, we noticed that KIRC patients with low ACO1 or IREB2 contents exhibited a remarkably worse survival rate in contrast with those with high expression in Kaplan–Meier survival analyses **(** **Figure 4** **)**. These conclusions validated that ACO1 and IREB2 may be novel independent predictive signatures in KIRC and may facilitate the occurrence and development of targeted precision oncology.

Autophagy constitutes a cellular degradation system that clears defective or superfluous proteins and organelles in cells, as well as acts as a substitute material and energy source during metabolic stress to keep cells alive. Autophagy also contributes to the maintenance of cellular physiological iron balance by assisting in the breakdown of the iron-storage protein ferritin (44). Through iron deprivation, autophagy coupled with mitophagy is activated in a variety of cell types (45–47). IRP1 detects the amounts of cellular iron and iron–sulfur clusters before fine-tuning the translational efficiency of Bcl-xL mRNA to control mitophagy activity (48). IRP1 is required for mitophagy triggered by iron stress. In response to iron deprivation, the deregulation of IRP1 disrupts iron-stress-driven mitophagy, resulting in an increase in mitochondrial ROS generation and oxidized mitochondrial proteins (48). Curcumin noticeably induced ferroptosis *via* activating autophagy in NSCLC, while the knockdown of IREB2 remarkably weakened curcumin-caused tumor suppressor ability in lung cancer cells (49). Consistently, in this study, ACO1 and IREB2 were tightly associated with autophagy probably through interaction with HIF-1A, which could induce mitochondrial autophagy and maintain cell survival (50).

Ferroptosis is a sort of planned cell death that occurs as a result of iron-dependent lipid peroxidation, as opposed to other types of cell death, such as autophagy (51). Until now, the influence of autophagy on ferroptosis remains unknown. Although new research shows that autophagy plays a role in ferroptotic cell death in breast cancer cells, as well as wild-type mouse embryonic fibroblasts, their findings are contradictory (29, 52–54). Gibson’s team thought that autophagy-triggered cell death occurred independently in breast cancer cells during ferroptosis (52). Autophagy, according to Jiang’s team, influences ferroptosis *via* modulating cellular iron homeostasis and cellular ROS formation (29). Hou’s team established that autophagy promotes ferroptosis by lowering ferritin contents (53). Tang’s team found that autophagy participates in curcumin-triggered NSCLC ferroptosis and that dampening autophagy reduced cell susceptibility to cellular ferroptosis (49). For ferroptotic cell death, different inducers and cell types may trigger distinct biological processes. This research confirms the concept that autophagy modulates ferroptosis in KIRC by modifying cellular iron homeostasis. The downregulation of ACO1 and IREB2 in KIRC fails to maintain cellular iron homeostasis probably *via* interaction with FTH1, HAMP, NCOA4, and HIF1A.

ACO1 and IREB2 are activated in response to the iron-deficient condition (8). A previous study reported that the viability of IRP1 binding to IRE was assessed, following immune induction, which was considered as part of the insect immune response (55). ACO1, as one of immune-related prognostic signatures, was used for foreseeing the prognosis of breast cancer or endometrial cancer patients (56, 57). The influence of iron availability to the parasite by IRP2 could be inhibited by IFN-γ and augmented by IL-10 (58), which plays an essential role in the modulation of inflammation along with the immune response. As far as we know, the network between ACO1, IREB2 and immune cell invasion in renal cancer has not been clearly studied. In current research, the phenomenon that ACO1 and IREB2 are involved in numerous cascades were revealed in GO along with the KEGG pathway enrichment analyses of ACO1, IREB2 and the linked genes, especially the PD-L1 expression state and PD-1 checkpoint cascade in cancer **(** **Figure 7** **)**. This finding was in line with the former literature, reinforcing the link between ACO1, IREB2 and the immune response. Here, we report a novel founding that low ACO1 and IREB2 contents in renal cancer are linked to the decreased invasion of B cells, neutrophils, CD4+ T cells, dendritic cells, and CD8+ T cells, along with macrophages **(** **Figure 7** **)**.

Furthermore, a noticeable relationship of ACO1, IREB2 with numerous immune cell signature sets was noticed in KIRC **(** **Supplemental Figures 5**–**7** **)**. ACO1 and IREB2 contents were also directly link with PD-1, PD-L1, CTLA-4, LAG3, and HAVCR2 contents **(** **Figure 7** **)**. More remarkably, ACO1 and IREB2 regulate the time of survival of individuals with renal cancer partly through immune cell invasion **(** **Figure 8** **)**. Our data support that ACO1 and IREB2 may be a novel powerful immune-linked treatment target in RCC. Insufficiently, the tangible role of ACO1 and IREB2 in the tumor-immune microenvironment still requires further in-depth investigation.

Even though current research forwards our understanding of the connection between ACO1, IREB2 and autophagy, ferroptosis, and immune infiltration in KIRC, some limitations still occur. First, there is an unavailability of interpretation of the mechanisms underlying autophagy-related ferroptosis induced by ACO1 and IREB2 even when we explored the correlation between ACO1, IREB2, and autophagy-related ferroptosis in KIRC patients. Second, we observed that ACO1 and IREB2 were weakly expressed in KIRC patients and the clinical association with immune infiltration. Still, further research into the apparent modulatory mechanisms along the functions of ACO1, IREB2 in tumor proliferation, metastasis, and immunological invasion is required. Third, in contemporary research, numerous studies were based on the mRNA contents of ACO1 and IREB2. A more thorough investigation, based on protein contents, might yield more persuasive results. Fourthly, we did not evaluate the diagnostic along with the prognostic worth of ACO1 and IREB2 in papillary RCC (PRCC), chromophobe RCC (CRCC), and other histological types of RCC in our research. Overall, our results confirm that ACO1 and IREB2 could act as a potential powerful prognostic biomarker for KIRC. Furthermore, we studied the underlying evidence pointing that ACO1 and IREB2 regulate autophagy-linked ferroptosis along with immune cell invasion in the TME in KIRC patients. Consequently, these findings own an underlying value in deepening our current comprehension of not only the characters of ACO1 and IREB2 but also their translational use in KIRC prognosis and targeted therapy.

For advanced RCC, multikinase repressors, for instance, sorafenib, are the first line of treatment (59). Strikingly, sorafenib accelerates hepcidin gene contents in Huh7 liver cancer cells when combined with numerous other kinase repressors, consisting of phosphoinositide 3-kinase (PI3K), the mechanistic target of rapamycin (mTOR), Ras/mitogen-activated protein kinase (MAPK), along with AMP-activated protein kinase (AMPK) cascade inhibitors (45). Herein, blocking the activation of ACO1 or IREB2 by its inhibitors OMA or SNP (39, 40) ameliorated sorafenib-triggered cell death, supporting that ACO1 and IREB2 could participate in its cytotoxic influence on renal cancer cells. Our gains were strongly confirmed by previous investigations using the IREB2 siRNA approach (38). It is integral to verify the possible significance of ACO1 and IREB2 contents as a powerful signature for targeted treatment or novel immunotherapy in clinical settings.

## Data availability statement

Publicly available datasets were analyzed in this study. This data can be found here:

http://timer.cistrome.org/

http://ualcan.path.uab.edu/analysis.html

http://www.proteinatlas.org/

http://www.xiantao.love

http://genemania.org/

http://gepia2.cancer-pku.cn/#index

http://www.autophagy.lu/index.html

http://www.zhounan.org/ferrdb/index.html

https://kmplot.com/analysis/

http://cis.hku.hk/TISIDB/index.php.

## Author contributions

LCD and ZT designed the study. ZJH, TH and YHY carried out the experiments of cytobiology. XZY, CTY and WHY conducted the follow-up study. CQ, YJK and ZQZ participated in acquisition of data. XKY, WHY and CMK took part in collection of clinical samples. GWB, XM and BJM analyzed the experimental data. ZJH drafted the manuscript. YC, DHF and HZP participated in the revising of the manuscript. All authors read and approved the final manuscript.

## Funding

This work was supported by National Natural Science Foundation of China (No. 81902991), Guangzhou Science and Technology Plan Project (No. 202102021056) and Youth Project of The Third Affiliated Hospital of Southern Medical University (No. QD2019N010).

## Conflict of interest

The authors declare that the research was conducted in the absence of any commercial or financial relationships that could be construed as a potential conflict of interest.

## Publisher’s note

All claims expressed in this article are solely those of the authors and do not necessarily represent those of their affiliated organizations, or those of the publisher, the editors and the reviewers. Any product that may be evaluated in this article, or claim that may be made by its manufacturer, is not guaranteed or endorsed by the publisher.
