# Supplementary material for: ACO1 and IREB2 downregulation confer poor prognosis and correlate with autophagy-related ferroptosis and immune infiltration in KIRC
Source: Front Oncol. 2022 Aug 17;12:929838. doi: 10.3389/fonc.2022.929838 (PMC9428356; doi:10.3389/fonc.2022.929838)
Supplement: Supplementary file 1 [file Table_1.docx]

| Supplemental Table 1. Clinical charateristics of the KIRC patients in TCGA database. | | |
| --- | --- | --- |
| Characteristic | levels | Overall |
| n |  | 539 |
| Age, n (%) | <=60 | 269 (49.9%) |
|  | >60 | 270 (50.1%) |
| Gender, n (%) | Female | 186 (34.5%) |
|  | Male | 353 (65.5%) |
| Race, n (%) | Asian | 8 (1.5%) |
|  | Black or African American | 57 (10.7%) |
|  | White | 467 (87.8%) |
| T stage, n (%) | T1 | 278 (51.6%) |
|  | T2 | 71 (13.2%) |
|  | T3 | 179 (33.2%) |
|  | T4 | 11 (2%) |
| N stage, n (%) | N0 | 241 (93.8%) |
|  | N1 | 16 (6.2%) |
| M stage, n (%) | M0 | 428 (84.6%) |
|  | M1 | 78 (15.4%) |
| Pathologic stage, n (%) | Stage I | 272 (50.7%) |
|  | Stage II | 59 (11%) |
|  | Stage III | 123 (22.9%) |
|  | Stage IV | 82 (15.3%) |
| Histologic grade, n (%) | G1 | 14 (2.6%) |
|  | G2 | 235 (44.3%) |
|  | G3 | 207 (39%) |
|  | G4 | 75 (14.1%) |
| Primary therapy outcome, n (%) | Progressive Disease | 11 (7.5%) |
|  | Stable Disease | 6 (4.1%) |
|  | Partial Response | 2 (1.4%) |
|  | Complete Response | 128 (87.1%) |
| Serum calcium, n (%) | Elevated | 10 (2.7%) |
|  | Low | 203 (55.5%) |
|  | Normal | 153 (41.8%) |
| Hemoglobin, n (%) | Elevated | 5 (1.1%) |
|  | Low | 263 (57.3%) |
|  | Normal | 191 (41.6%) |
| Laterality, n (%) | Left | 252 (46.8%) |
|  | Right | 286 (53.2%) |
| Overall Survival, n (%) | Alive | 366 (67.9%) |
|  | Dead | 173 (32.1%) |
| Disease Specific Survival, n (%) | Alive | 420 (79.5%) |
|  | Dead | 108 (20.5%) |
| Progress Free Interval, n (%) | Alive | 378 (70.1%) |
|  | Dead | 161 (29.9%) |
