# Supplementary material for: ACO1 and IREB2 downregulation confer poor prognosis and correlate with autophagy-related ferroptosis and immune infiltration in KIRC
Source: Front Oncol. 2022 Aug 17;12:929838. doi: 10.3389/fonc.2022.929838 (PMC9428356; doi:10.3389/fonc.2022.929838)
Supplement: Supplementary file 2 [file Table_2.docx]

| Characteristics | Total(N) | Univariate analysis | |  | Multivariate analysis | |
| --- | --- | --- | --- | --- | --- | --- |
|  |  | Hazard ratio (95% CI) | P value |  | Hazard ratio (95% CI) | P value |
| Age | 539 |  |  |  |  |  |
| <=60 | 269 | Reference |  |  |  |  |
| >60 | 270 | 1.765 (1.298-2.398) | **<0.001** |  | 1.445 (0.931-2.242) | 0.101 |
| Gender | 539 |  |  |  |  |  |
| Female | 186 | Reference |  |  |  |  |
| Male | 353 | 0.930 (0.682-1.268) | 0.648 |  |  |  |
| T stage | 539 |  |  |  |  |  |
| T1&T2 | 349 | Reference |  |  |  |  |
| T3&T4 | 190 | 3.228 (2.382-4.374) | **<0.001** |  | 1.531 (0.667-3.515) | 0.315 |
| N stage | 257 |  |  |  |  |  |
| N0 | 241 | Reference |  |  |  |  |
| N1 | 16 | 3.453 (1.832-6.508) | **<0.001** |  | 1.519 (0.754-3.061) | 0.242 |
| M stage | 506 |  |  |  |  |  |
| M0 | 428 | Reference |  |  |  |  |
| M1 | 78 | 4.389 (3.212-5.999) | **<0.001** |  | 2.760 (1.632-4.669) | **<0.001** |
| ACO1 | 539 |  |  |  |  |  |
| Low | 269 | Reference |  |  |  |  |
| High | 270 | 0.620 (0.457-0.842) | **0.002** |  | 0.598 (0.362-0.987) | **0.044** |
| IREB2 | 539 |  |  |  |  |  |
| Low | 269 | Reference |  |  |  |  |
| High | 270 | 0.535 (0.392-0.731) | **<0.001** |  | 0.866 (0.525-1.429) | 0.573 |
| Pathologic stage | 536 |  |  |  |  |  |
| Stage I&Stage II | 331 | Reference |  |  |  |  |
| Stage III&Stage IV | 205 | 3.946 (2.872-5.423) | **<0.001** |  | 1.227 (0.480-3.137) | 0.670 |
| Histologic grade | 531 |  |  |  |  |  |
| G1&G2 | 249 | Reference |  |  |  |  |
| G3&G4 | 282 | 2.702 (1.918-3.807) | **<0.001** |  | 1.726 (1.037-2.872) | **0.036** |
