# Supplementary figures and images for: ACO1 and IREB2 downregulation confer poor prognosis and correlate with autophagy-related ferroptosis and immune infiltration in KIRC
Source: Front Oncol. 2022 Aug 17;12:929838. doi: 10.3389/fonc.2022.929838 (PMC9428356; doi:10.3389/fonc.2022.929838)

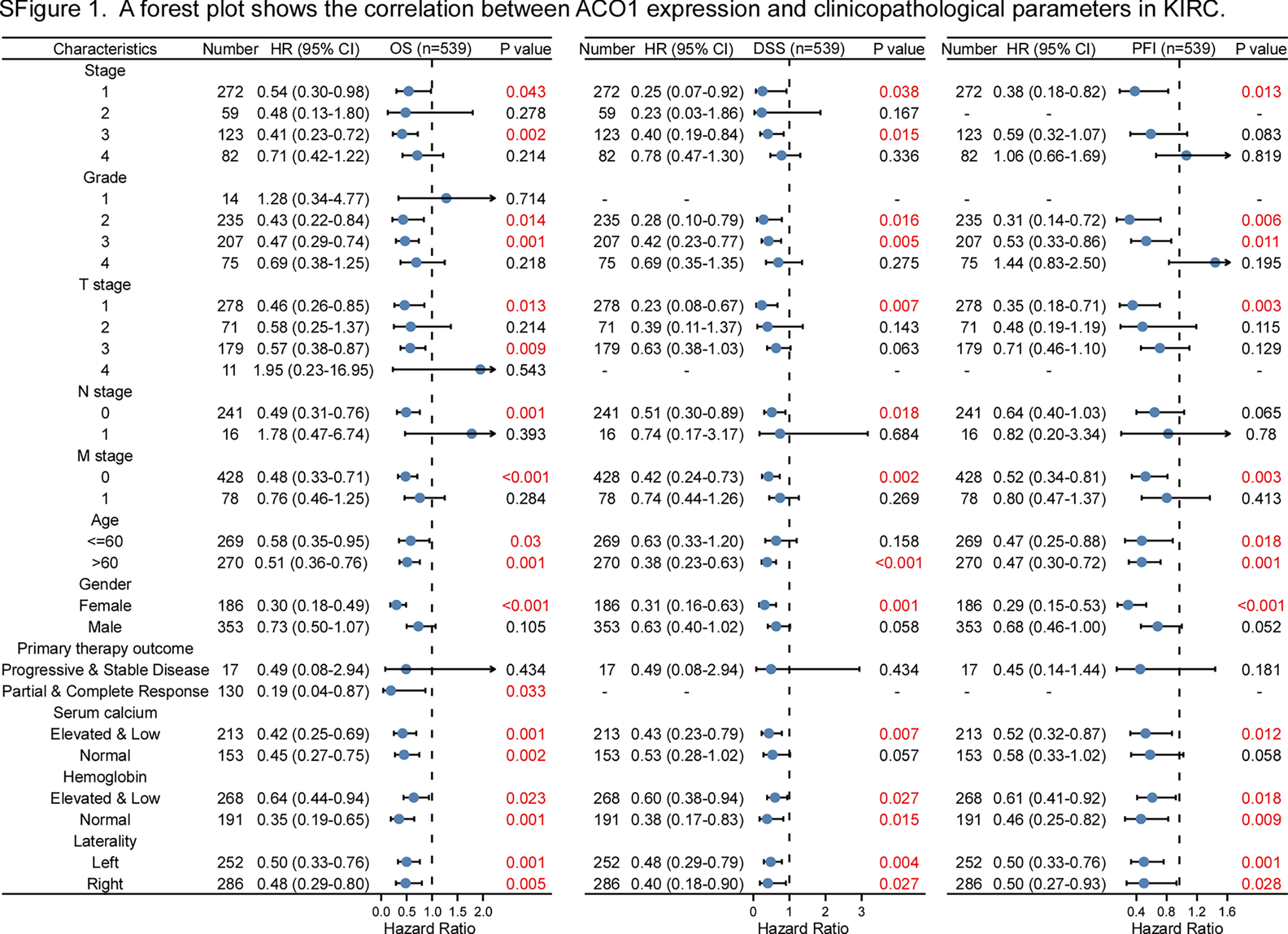

Supplement: Supplementary file 3 [file Image_1.tiff]

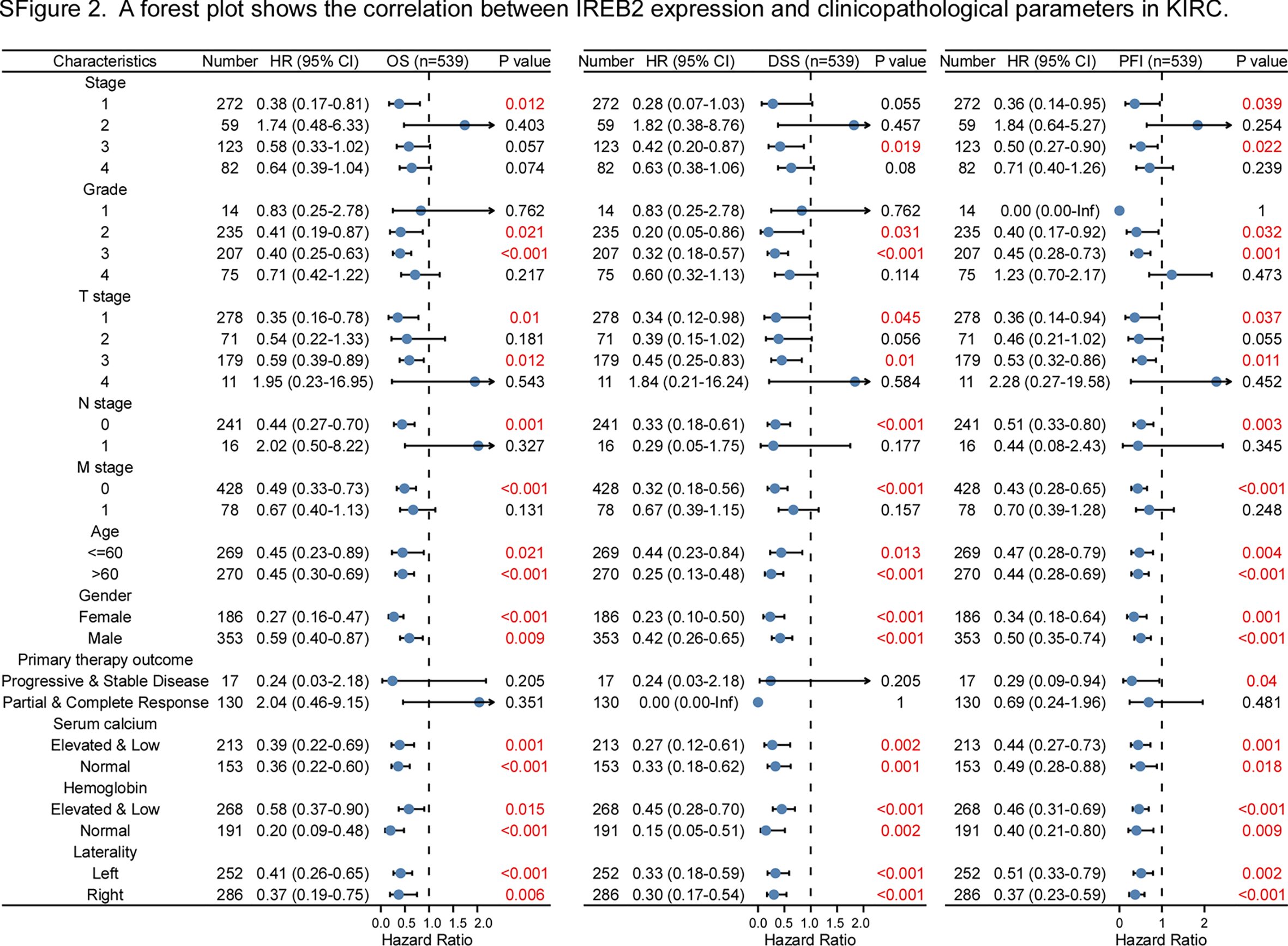

Supplement: Supplementary file 4 [file Image_2.tiff]

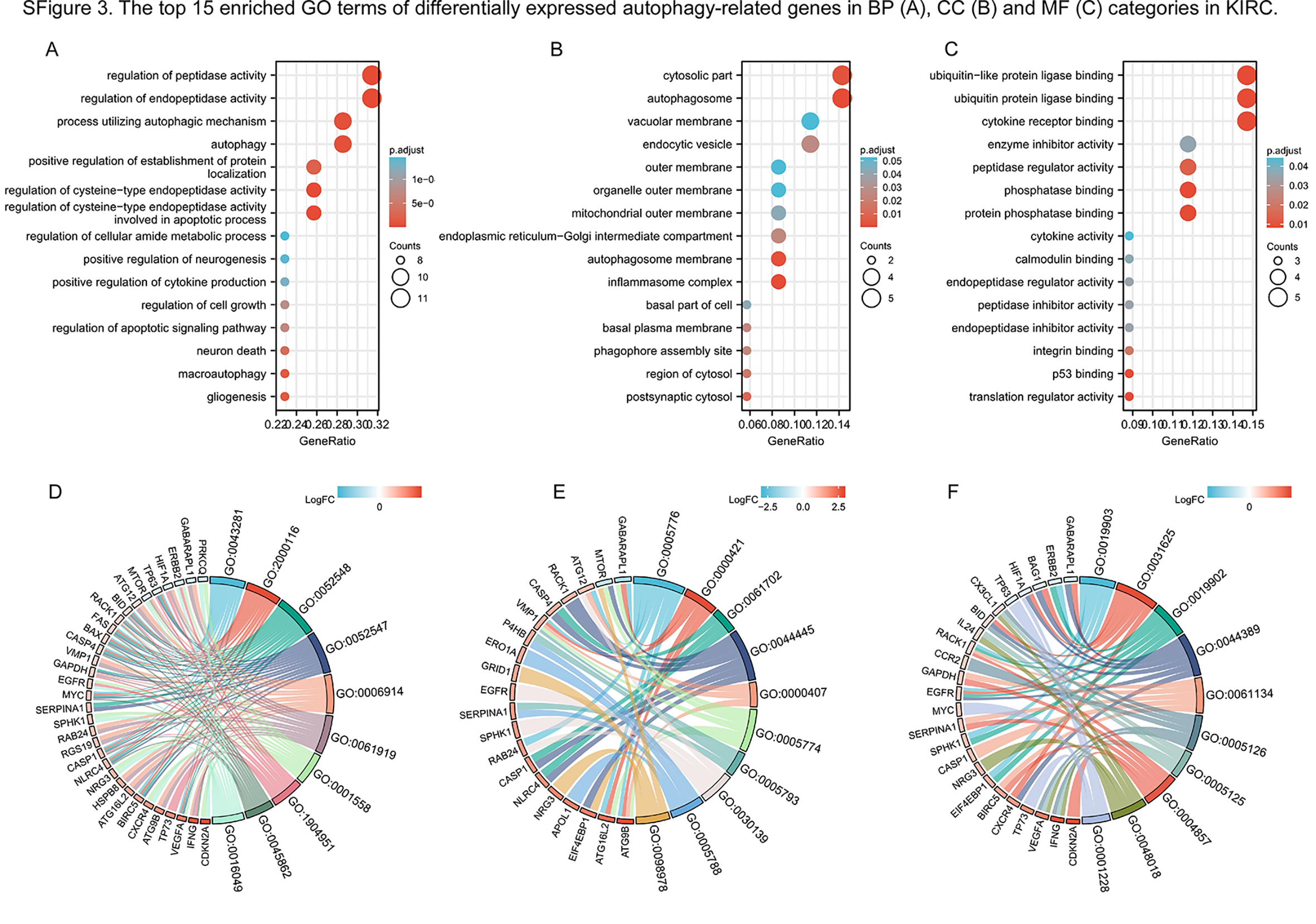

Supplement: Supplementary file 5 [file Image_3.tiff]

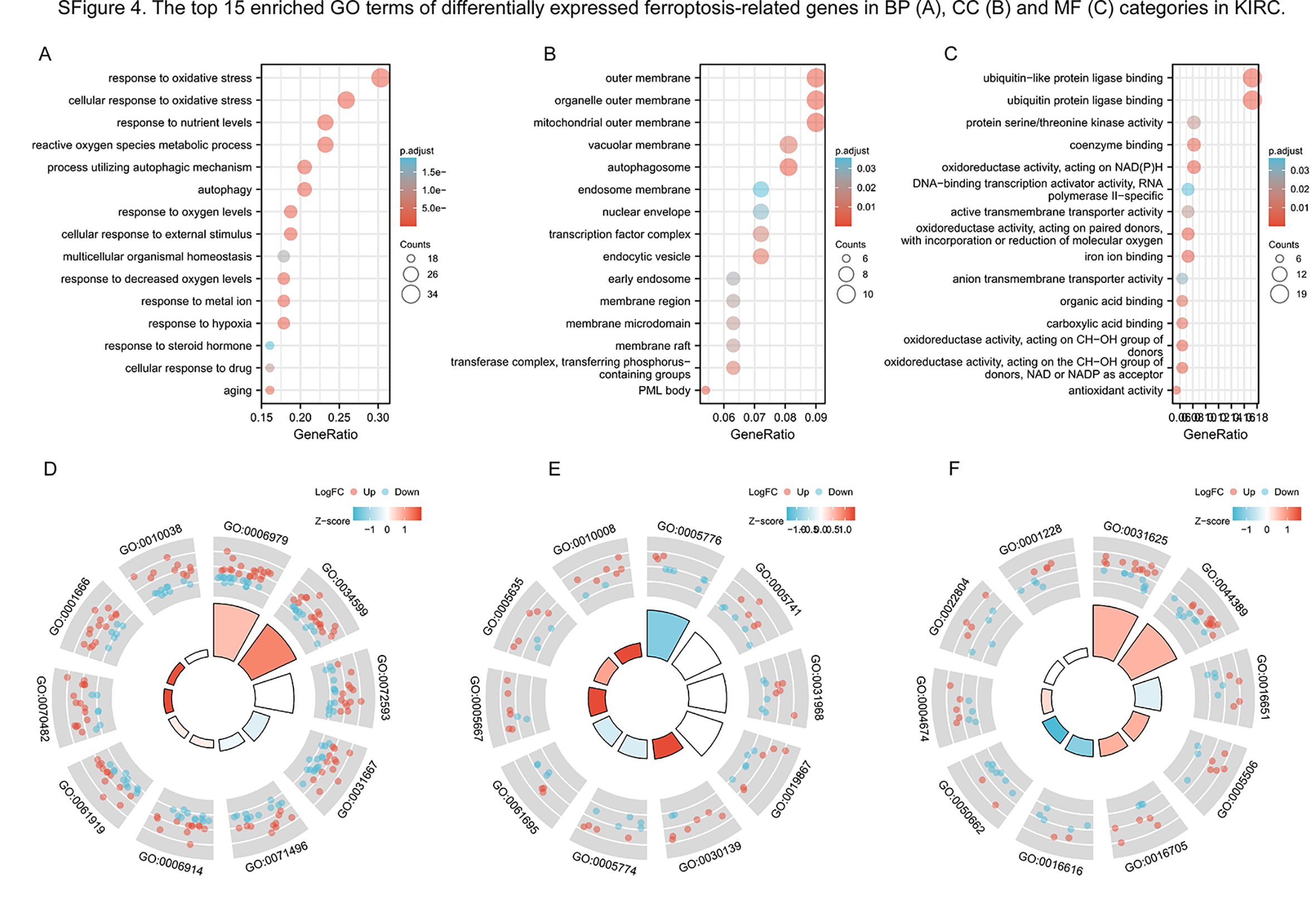

Supplement: Supplementary file 6 [file Image_4.tiff]

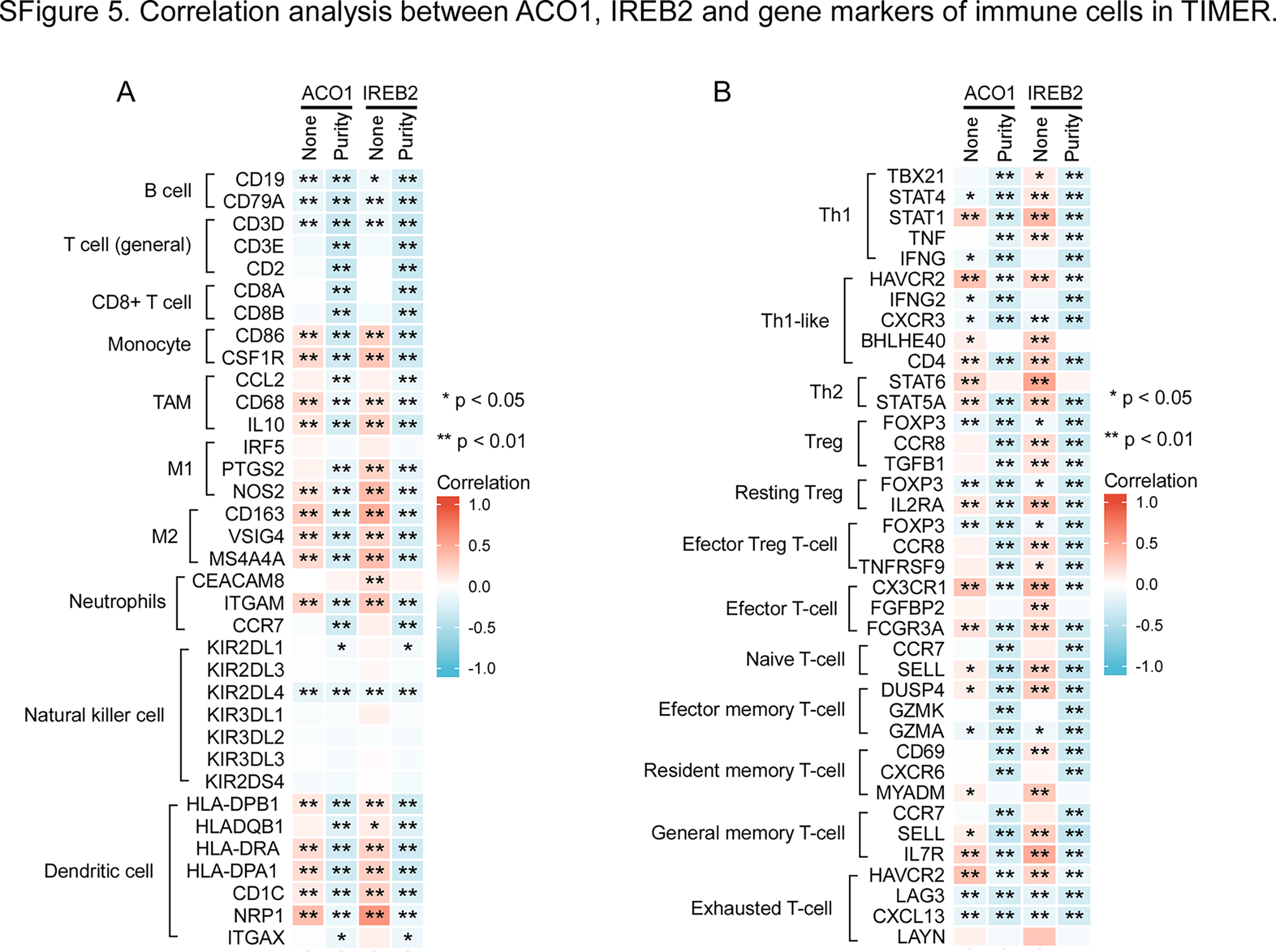

Supplement: Supplementary file 7 [file Image_5.tiff]

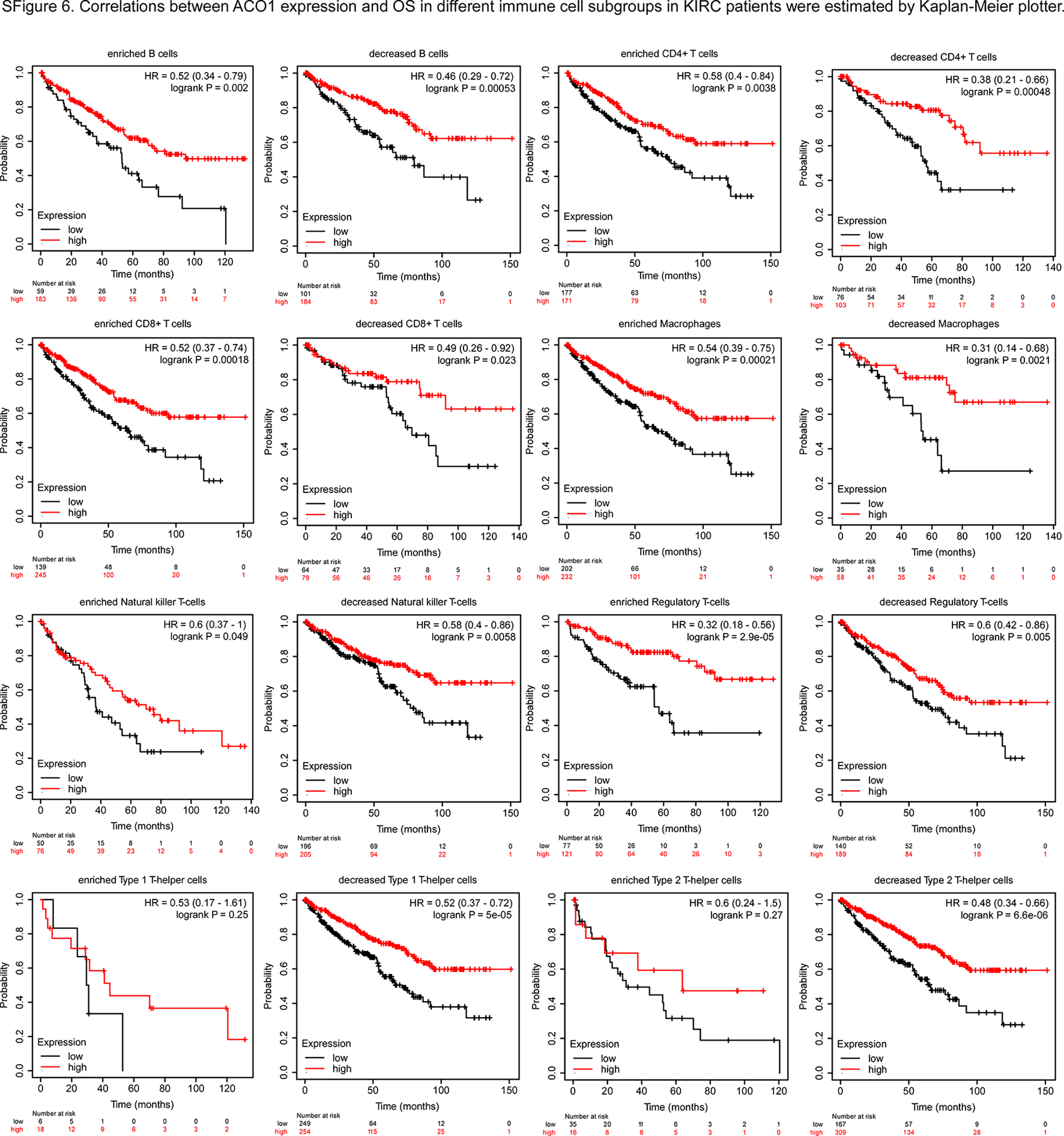

Supplement: Supplementary file 8 [file Image_6.tiff]

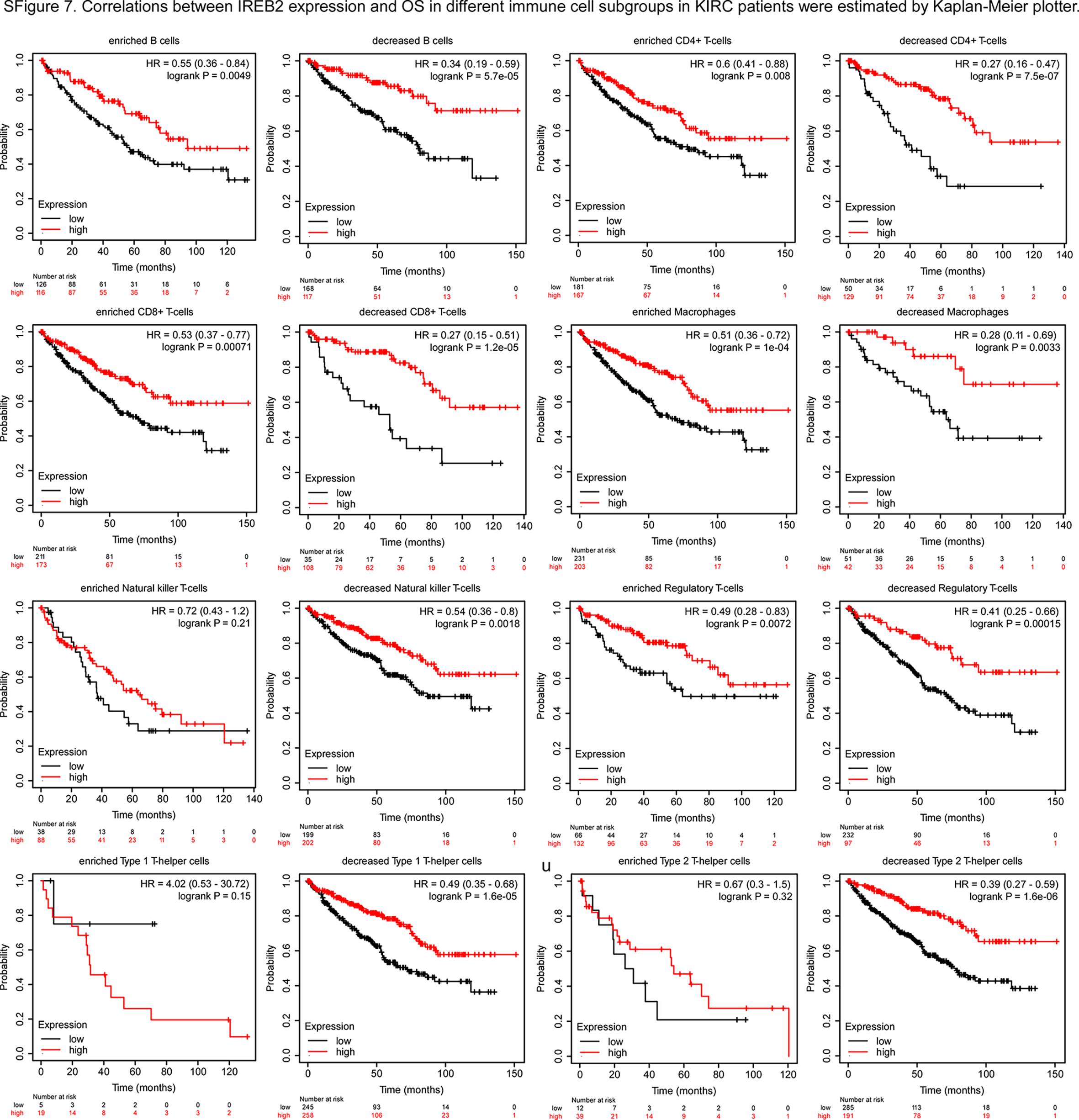

Supplement: Supplementary file 9 [file Image_7.tiff]

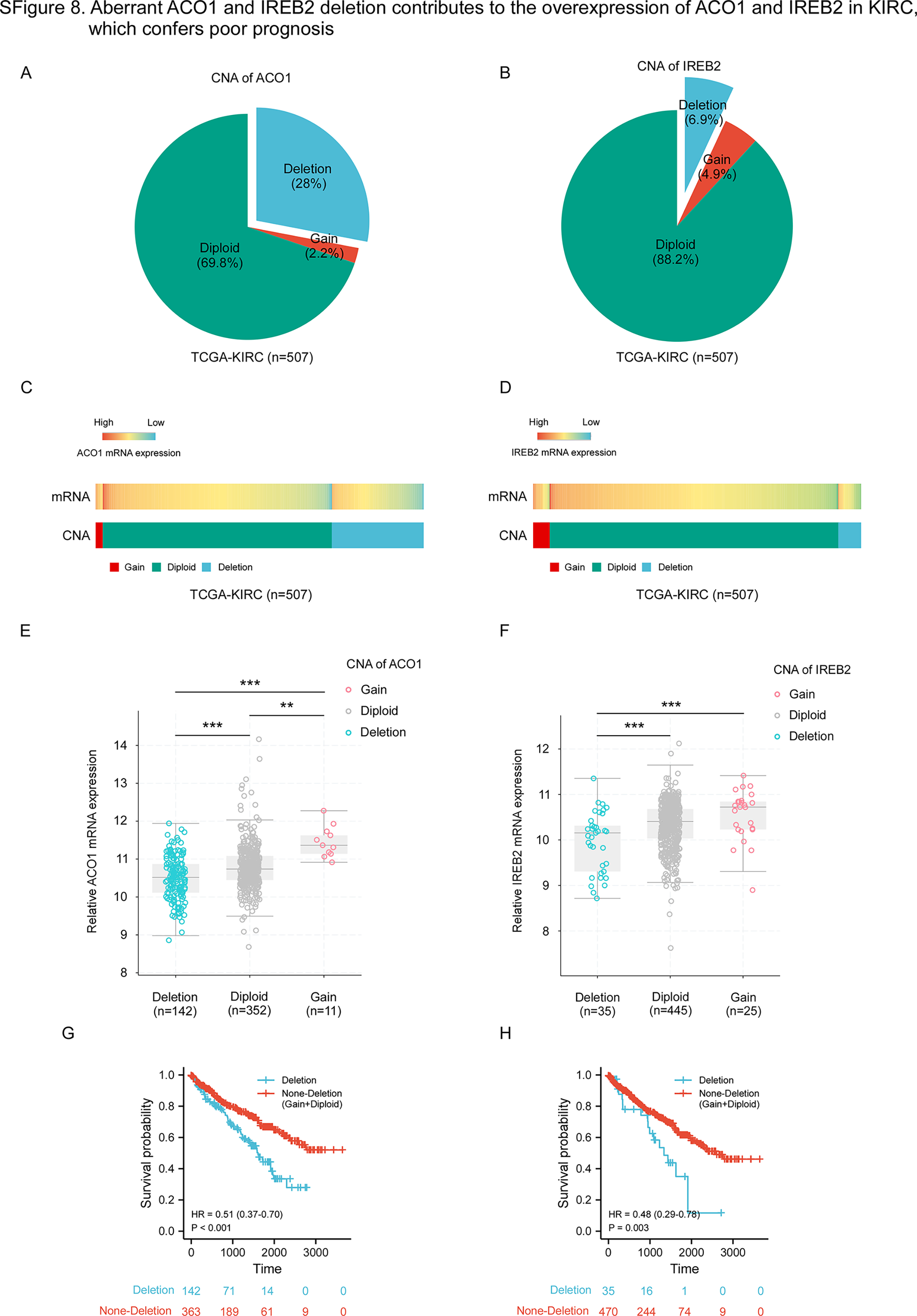

Supplement: Supplementary file 10 [file Image_8.tiff]

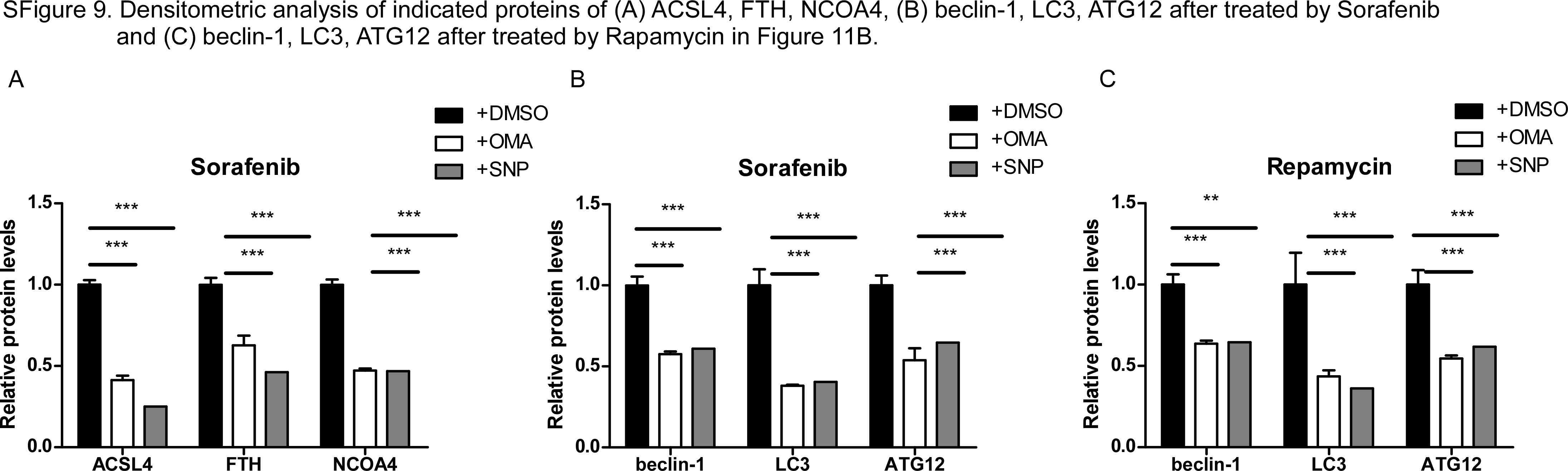

Supplement: Supplementary file 11 [file Image_9.tiff]
